# The maternal microbiome promotes placental development in mice

**DOI:** 10.1126/sciadv.adk1887

**Published:** 2023-10-06

**Authors:** Geoffrey N. Pronovost, Kristie B. Yu, Elena J. L. Coley-O’Rourke, Sahil S. Telang, Angela S. Chen, Helen E. Vuong, Drake W. Williams, Anisha Chandra, Tomiko K. Rendon, Jorge Paramo, Reuben H. Kim, Elaine Y. Hsiao

**Affiliations:** ^1^ Department of Integrative Biology and Physiology, University of California, Los Angeles, Los Angeles, CA, USA.; ^2^ The Shapiro Family Laboratory of Viral Oncology and Aging Research, University of California, Los Angeles, Los Angeles, CA, USA.; ^3^ UCLA Goodman-Luskin Microbiome Center, Division of Digestive Diseases, Department of Medicine, David Geffen School of Medicine, Los Angeles, CA, USA.

## Abstract

The maternal microbiome is an important regulator of gestational health, but how it affects the placenta as the interface between mother and fetus remains unexplored. Here, we show that the maternal gut microbiota supports placental development in mice. Depletion of the maternal gut microbiota restricts placental growth and impairs feto-placental vascularization. The maternal gut microbiota modulates metabolites in the maternal and fetal circulation. Short-chain fatty acids (SCFAs) stimulate cultured endothelial cell tube formation and prevent abnormalities in placental vascularization in microbiota-deficient mice. Furthermore, in a model of maternal malnutrition, gestational supplementation with SCFAs prevents placental growth restriction and vascular insufficiency. These findings highlight the importance of host-microbial symbioses during pregnancy and reveal that the maternal gut microbiome promotes placental growth and vascularization in mice.

## Teaser

The maternal gut microbiome and SCFAs promote placental growth and vascularization in gnotobiotic and malnourished mice.

## INTRODUCTION

Recent studies highlight notable influences of the maternal microbiome on offspring development that begin during the prenatal period (*1*, *2*), but exactly how the maternal microbiome informs maternal-fetal health during pregnancy remains unclear. At the intersection of mother and fetus is the highly vascularized placenta, which is responsible for enabling maternal-fetal exchange of nutrients and gases that sustain fetal development (*3*, *4*). We examined effects of the maternal gut microbiome on the development of the placenta in mice, as a critical organ that shapes long-term health trajectories.

## RESULTS

**Fig. 1.**
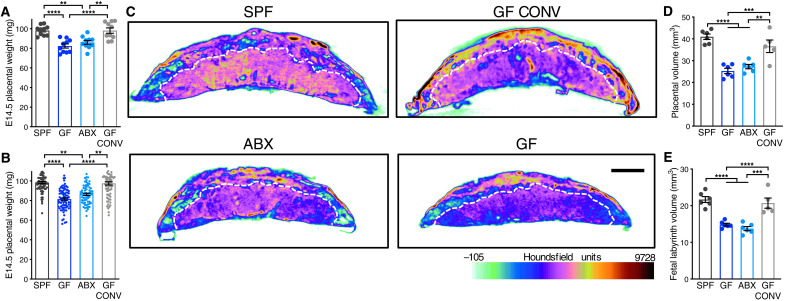
The maternal microbiome promotes placental development. (**A**) E14.5 placental weights by litter average [SPF (*n* = 10), GF (*n* = 10), ABX (*n* = 10), and GF CONV (*n* = 10)]. (**B**) E14.5 placental weights for each individual from litters shown in Fig. 1A [SPF (*n* = 80), GF (*n* = 81), ABX (*n* = 65), and GF CONV (*n* = 77)]. (**C**) Representative cross sections of E14.5 whole-placental μCT reconstructions from SPF, GF, ABX, and GF CONV litters. White hashed line distinguishes fetal placental labyrinth compartment; scale bar, 1 mm; Houndsfield scale ranges from −105 (light green) to 9728 (maroon). (**D**) Quantification of E14.5 whole-placental volumes from μCT reconstructions by litter average [SPF (*n* = 6), GF (*n* = 6), ABX (*n* = 6), and GF CONV (*n* = 5)]. (**E**) Quantification of fetal placental labyrinth volumes from μCT reconstructions shown in (D). Data represent mean ± SEM; statistics were performed with one-way ANOVA with Tukey post hoc test (litter averages) or with one-way nested with Sidak multiple comparisons correction (individual conceptuses). **P < 0.01, ***P < 0.001, and ****P < 0.0001.

To determine effects of the maternal gut microbiome on placental development, we first reared pregnant mice as germ-free (GF) or depleted the maternal gut microbiome by treating with broad-spectrum antibiotics (ABX). Absence or depletion of the maternal gut microbiome resulted in reduced placental weight at embryonic day 14.5 (E14.5), relative to conventionally colonized [specific pathogen-free (SPF)] and conventionalized GF controls (i.e., GF mice colonized with SPF microbiota during adulthood, GF CONV) (Fig. 1, A and B). Maternal treatment with the subset of ABX that are nonabsorbable phenocopied the reduced placental weight seen with the full ABX cocktail (fig. S1, A to D), with expected reductions in microbial diversity (fig. S1, E to G). This suggests that ABX-induced reductions in placental weight are due to depletion of the maternal gut microbiome rather than off-target effects of ABX. To determine whether changes in placental weight are due to localized disruptions to particular subregions of the placenta, placentas from gnotobiotic dams were imaged by micro–computed tomography (μCT). We considered all tissue density detected by μCT for volumetric analyses and applied colorimetric overlays of Houndsfield units, a measure of radiodensity, to display relative changes in subregion tissue density. Consistent with reductions in placental weight, maternal microbiome deficiency led to reductions in total placental volume, along with reduced volume and tissue density in the placental labyrinth, the primary site for maternal-fetal exchange (Fig. 1, C to E). In addition to maternal ABX-induced placental pathophysiology, we observed corresponding decreases in fetal weight and volume on E14.5, which were not seen in the GF condition (fig. S2). Reductions in fetal weight were similarly apparent on E18.5 (fig. S3), indicating that maternal microbiome depletion induces persistent impairments in fetal growth. However, we observed no significant differences between groups in placental weight on E18.5 (fig. S3), which were driven by expected reductions in total placental size with advanced gestational age (*5*) in SPF and GF CONV conditions and lack of change in GF and ABX conditions. Overall, these data reveal a key role for the maternal gut microbiome in promoting placental growth and development, in particular within the labyrinth subregion.

**Fig. 2.**
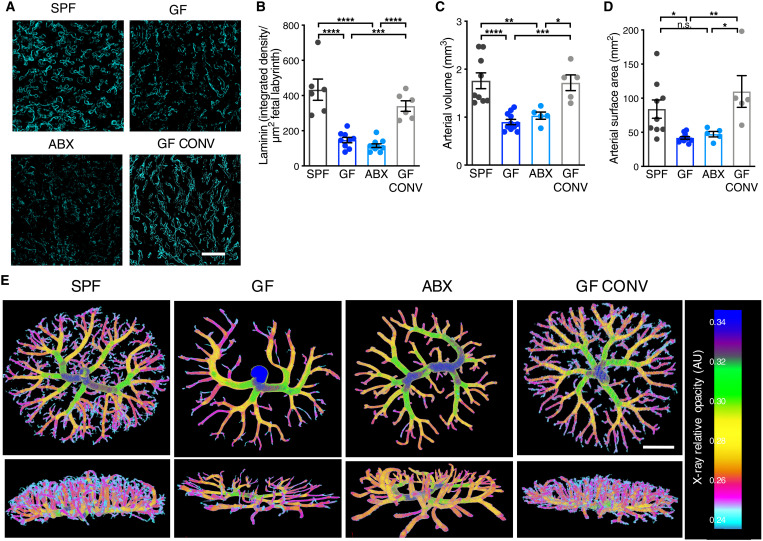
The maternal microbiome promotes placental vascular development. (**A**) Representative images of laminin-stained E14.5 placental labyrinth from SPF, GF, ABX, and GFCONV litters (scale bar, 100 μm). (**B**) Quantification of raw integrated density of laminin staining intensity, normalized to fetal labyrinth total area [SPF (*n* = 6), GF (*n* = 9), ABX (*n* = 10), and GF CONV (*n* = 5)]. (**C**) Quantification of E14.5 feto-placental arterial vascular volume [SPF (*n* = 9), GF (*n* = 11), ABX (*n* = 5), and GF CONV (*n* = 5)]. (**D**) Quantification of E14.5 feto-placental arterial vascular surface area [SPF (*n* = 9), GF (*n* = 11), ABX (*n* = 5), and GF CONV (*n* = 5)]. (**E**) Representative feto-placental arterial vascular reconstructions by μCT imaging of vascular casts from E14.5 SPF, GF, ABX, and GF CONV litters; scale bar, 1 mm. Data represent mean ± SEM; statistics were performed with one-way ANOVA with Tukey post hoc test. *P < 0.05, **P < 0.01, ***P < 0.001, and ****P < 0.0001.

The placental labyrinth is composed of maternal and fetal blood spaces separated by trophoblast cells, basement membrane, and fetal endothelial cells that together mediate gas and nutrient exchange. Following our observations of reduced placental labyrinth volume, and considering the coincident rapidly growing fetal placental vasculature during mid-gestation (*6*, *7*), we hypothesized that maternal microbiota depletion could alter development of feto-placental vasculature. To test this, we stained placentas with laminin to visualize the extracellular matrix underlying placental endothelial cells and saw significantly reduced staining intensity in placental labyrinth tissue from microbiome-deficient dams relative to controls (Fig. 2, A to B). We further generated feto-placental arterial casts and analyzed their native structures using μCT imaging. Feto-placental vasculature from microbiota-deficient dams exhibited reduced vascular volume and surface area on E14.5, with visible decreases in vascular branches, as compared to controls (Fig. 2, C to E). This aligns with prior studies that have reported a role for the microbiome in regulating angiogenesis and barrier integrity in other organs, including the intestine and brain (*8*, *9*). There was a higher variance in data for the SPF groups compared to both the GF and ABX groups. The variance seen in SPF groups aligns with those previously reported for conventionally colonized controls (*10*) and could reflect natural biological variation seen between different placentae within the same litter and/or expected biological variation seen across different litters for which the same gestational age is used to refer to conceptuses that differ by up to 12 hours in mouse development. The observed reduction in feto-placental vascular branches in midgestational placentas from microbiota-deficient dams suggests that the maternal microbiome instructs vascular development at critical gestational time points. To gain insight into whether microbiome-dependent disruptions in placental physiology are detectable at earlier gestational ages and persistent through late gestation, we followed by assessing placental vasculature at E10.5 and E18.5. We did not observe maternal microbiome-dependent differences in labyrinth endothelial CD31 staining of E10.5 litters (fig. S4) or laminin staining of E18.5 litters (fig. S5, A to B), despite an overall reduction in feto-placental arterial volume in E18.5 GF and ABX litters (fig. S5, C to E), suggesting that effects of the maternal microbiome are most apparent during midgestation, a time of rapid placental growth. Collectively, these data reveal that the maternal microbiome is required for proper development of feto-placental vasculature.

Given that the maternal microbiome regulates many circulating metabolites (*2*), we postulated that microbiome-dependent insufficiencies in feto-placental vasculature could arise from alterations in key metabolites in the fetal circulation. Therefore, we performed untargeted metabolomic profiling of 753 metabolites in the fetal serum metabolome (table S1). By principal components analysis, fetal serum metabolomic profiles from GF and ABX dams generally clustered away from SPF controls (fig. S6A). On the basis of the shared placental deficiencies across both GF and ABX conditions, we then filtered for common metabolomic alterations. When applying a threshold of *P* < 0.05, 27 metabolites were commonly and significantly down-regulated and 14 were commonly and significantly up-regulated in fetal serum from GF and ABX dams, compared to SPF controls (fig. S6, B to D). Random forest analysis of the full metabolomic dataset identified 30 fetal serum metabolites that predicted maternal microbiota status with 89% accuracy (fig. S6E). While deficient vasculature may result in impaired nutrient and gas exchange, no statistically significant changes were seen for fetal serum metabolites related to core metabolism, including glucose and many amino acids (table S1), suggesting that the placental and fetal deficits caused by maternal microbiome deficiency are not due to overt fetal nutrient restriction. These data indicate that the maternal microbiome modulates the bioavailability of metabolites in the fetal circulation, which is consistent with previous data demonstrating that the maternal microbiome regulates metabolites in the maternal serum and fetal brain during pregnancy (*2*). To determine whether select maternal microbiome-dependent metabolites in the fetal serum promote feto-placental vascular development and placental growth, we supplemented microbiota-deficient dams with a pool of 19 metabolites that were significantly and commonly reduced in both maternal serum and fetal serum from microbiota-deficient dams (fig. S6, F to J). Daily systemic injection of the microbially modulated metabolites at physiologically relevant concentrations failed to prevent maternal ABX-induced reductions in placental and fetal weight (fig. S7, A to C) and in microvascular laminin staining of placental tissues (fig. S7, D and E). Thus, the placental insufficiencies induced by maternal microbiome deficiency are likely not regulated by this select group of maternal microbiome-dependent metabolites.

**Fig. 3.**
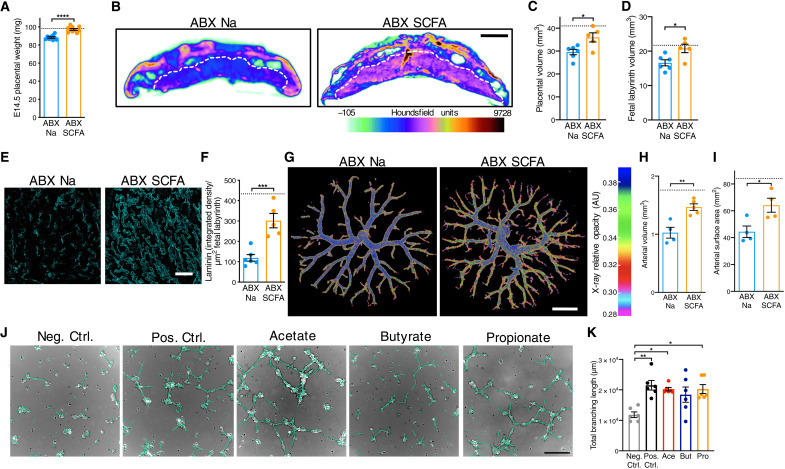
Maternal SCFA supplementation prevents impairments in placental growth and microvasculature induced by maternal microbiome depletion. (**A**) E14.5 placental weights by litter average [ABX Na (*n* = 9) and ABX SCFA (*n* = 10); hashed line represents SPF average from Fig. 1A]. (**B**) Cross sections of E14.5 whole-placental μCT reconstructions from ABX Na and ABX SCFA litters. (**C**) E14.5 whole-placental volumes from μCT reconstructions by litter average [ABX Na (*n* = 6) and ABX SCFA (*n* = 5); hashed line represents SPF average from Fig. 1D]. (**D**) Fetal placental labyrinth volumes from μCT reconstructions shown in (D); hashed line represents SPF average value from Fig. 1E. (**E**) Laminin-stained E14.5 placental labyrinth from ABX Na and ABX SCFA litters; scale bar, 100 μm. (**F**) Raw integrated density of laminin staining, normalized to fetal labyrinth total area [ABX Na (*n* = 6) and ABX SCFA (*n* = 5); hashed line represents SPF average from Fig. 2C]. (**G**) Representative feto-placental arterial vascular reconstructions from E14.5 ABX Na and ABX SCFA litters; scale bar, 1 mm. (**H**) E14.5 feto-placental arterial vascular volume [ABX Na (*n* = 4) and ABX SCFA (*n* = 4); hashed line represents SPF average from Fig. 2D]. (**I**) E14.5 feto-placental arterial vascular surface area from litters shown in (H); hashed line represents SPF average shown in Fig. 2E. (**J**) HUVEC tube formation assays (scale bar, 250 μm), depicting negative control (no supplementation), positive control (2% FBS supplementation), acetate supplementation (Ace, 40 μm), butyrate supplementation (But, 5 μm), and propionate supplementation (Pro, 5 μm). (**K**) HUVEC tube formation assays (*n* = 6 independent experiments). Data represent mean ± SEM; statistics were performed with Student’s *t* test or one-way ANOVA with Tukey post hoc test. *P < 0.05, **P < 0.01, ***P < 0.001, and ****P < 0.0001.

In addition to the metabolites identified and tested, we considered the role that microbial generation of short-chain fatty acids (SCFAs) might play in regulating placental and fetal development (*11*–*13*). SCFAs are produced by bacterial carbohydrate fermentation and are significantly decreased in the maternal and fetal serum from microbiota-deficient dams (*14*). On the basis of previous research demonstrating that maternal supplementation with SCFAs leads to direct transfer of SCFAs from maternal circulation to fetal circulation (*14*), we treated ABX dams with SCFA-supplemented water (*13*) or vehicle (sodium-matched) control water from E0.5 to E14.5. We confirmed that this supplementation strategy significantly increased butyrate and propionate concentrations in E14.5 fetal whole blood, with no change in SCFA levels in maternal serum or whole placental tissues at 1 hour after administration (fig. S8). This discrepancy may be due to pharmacokinetic distribution of SCFAs relative to time of consumption of the SCFA-supplemented water, as drinking cyclic and occurs mostly during the night in mice. Maternal SCFA treatment increased placental weight from ABX dams to levels comparable to SPF controls, with corresponding increases in total placental and labyrinth volumes as measured by μCT imaging (Fig. 3, A to D). Maternal SCFA treatment did not increase fetal weights from microbiota-depleted dams (fig. S9). Consistent with the increases in placental growth, maternal SCFA treatment increased endothelial laminin staining in placental labyrinth from ABX dams toward levels seen in SPF controls (Fig. 3, E and F). These observations were further supported using μCT reconstructions of feto-placental arterial casts, where maternal SCFA treatment increased fetal vascular volume and surface area in placentas from ABX dams (Fig. 3, G to I). Last, microbiota-depleted dams exhibited increased peak systolic velocity in the E14.5 placental labyrinth, aligning with responses to vasoconstriction, which was partially restored following SCFA treatment (fig. S10). There were no significant differences in peak systolic velocity detected in umbilical vessels (fig. S10). Given that umbilical flow increases in direct proportion to fetal weight, this could reflect an abnormal response and contributor to the significantly reduced size of ABX fetuses (fig. S2). Together, these data reveal that microbiota-derived SCFAs promote placental growth and feto-placental vascular development.

SCFAs have been reported to protect against aortic endothelial dysfunction and promote fibrovascular angiogenesis through direct receptor-mediated signaling (*15*, *16*). To gain insight into whether SCFAs may signal directly to fetal endothelial cells to promote vascularization, we treated human umbilical vein endothelial cells (HUVECs) with SCFAs at physiological concentrations (*17*, *18*) and quantified branching tube formation. The SCFAs acetate and propionate significantly increased HUVEC branching length compared to vehicle controls, whereas butyrate elicited variable increases in branching length that did not meet statistical significance (Fig. 3, J and K, and fig. S11). CRISPR-Cas9–mediated knockout of either free fatty acid receptor 2 [FFAR2; G-protein-coupled receptor 43 (GPR43)] or FFAR3 (GPR41) in HUVECs prevented the SCFA-induced increases in branching length, suggesting that direct signaling of SCFAs through both cognate receptors is required to stimulate tube formation (fig. S11G). Increased branching tube formation was not seen when cells were treated with various other microbiome-dependent metabolites (fig. S12), which were included in the pool that failed to prevent placental disruptions when administered to ABX dams (Fig. 3), suggesting specificity to SCFAs. Overall, these data indicate that SCFAs directly stimulate vascularization of cultured umbilical vein endothelial cells, consistent with our findings that SCFA supplementation promotes placental vascular development in vivo.

**Fig. 4.**
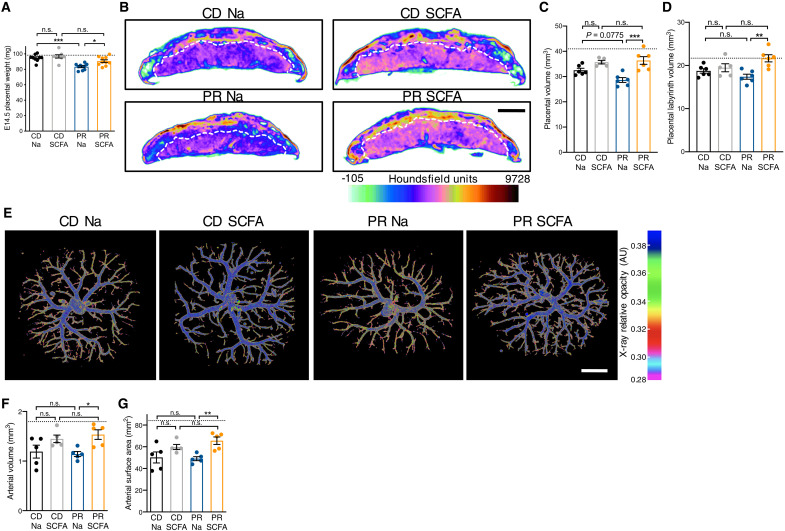
Maternal SCFA supplementation promotes placental growth and vascularization in protein-restricted dams. (**A**) E14.5 placental weights by litter average [CD Na (*n* = 9), CD SCFA (*n* = 10), PR Na (*n* = 8), and PR SCFA (*n* = 10); hashed line represents SPF litter average value shown in Fig. 1A]. (**B**) Representative cross sections of E14.5 whole-placental μCT reconstructions from CD Na, CD SCFA, PR Na, and PR SCFA litters. (**C**) Quantification of E14.5 whole-placental volumes from μCT reconstructions by litter average [CD Na (*n* = 6), CD SCFA (*n* = 5), PR Na (*n* = 6), and PR SCFA (*n* = 5); hashed line represents SPF litter average value shown in Fig. 1D]. (**D**) Quantification of fetal placental labyrinth volumes from μCT reconstructions shown in (C); hashed line represents SPF litter average value shown in Fig. 1E. (**E**) Representative feto-placental arterial vascular reconstructions by μCT imaging of vascular casts from CD Na, CD SCFA, PR Na, and PR SCFA litters; scale bar, 1 mm. (**F**) Quantification of E14.5 feto-placental arterial vascular volume [CD Na (*n* = 5), CD SCFA (*n* = 5), PR Na (*n* = 5), and PR SCFA (*n* = 5); hashed line represents SPF litter average value shown in Fig. 2D]. (**G**) Quantification of E14.5 feto-placental arterial surface area from litters shown in (F); hashed line represents SPF litter average value shown in Fig. 2D. Data represent mean ± SEM; statistics were performed with two-way ANOVA with Tukey post hoc test. *P < 0.05, **P < 0.01, and ***P < 0.001.

Maternal malnutrition, including maternal protein restriction (PR), is associated with placental insufficiencies, including reduced size and impaired vasculature (*19*, *20*). On the basis of the phenotypic similarities we observed in placentas from dams with deficient microbiomes (Figs. 1 to 3), we next asked whether maternal SCFA treatment could prevent placental abnormalities caused by maternal PR during pregnancy. To isolate effects of PR to restrictions in protein, rather than compensatory increases in complex carbohydrates, the PR diet and a control diet (CD) with levels of protein matching those in standard mouse chow were formulated with cellulose as the primary carbohydrate source (table S5). Consistent with previous findings (*20*), maternal PR reduced placental weight and volume, when compared to dams fed CD (Fig. 4, A to D). Maternal SCFA treatment was sufficient to restore placental weight, total volume, and labyrinth-specific volume in litters from PR dams to levels seen in CD and previous SPF controls (Fig. 4, A to D). Maternal SCFA treatment increased feto-placental vascularization, as measured by increases in feto-placental arterial vascular volume and surface area (Fig. 4, E to G). Fetal weights were neither affected by maternal diet nor SCFA treatment (fig. S13). Notably, we observed reductions in CD placental vasculature relative to SPF controls fed with diets rich in fiber, further suggesting the importance of SCFAs in promoting placental vasculature. However, PR did not induce alterations in maternal SCFA levels when compared to CD controls, suggesting that PR causes placental impairments through mechanisms that do not involve maternal SCFA deficiency (fig. S14). Together, these data suggest that SCFAs promote placental growth and vascular development, even under conditions of maternal malnutrition.

## DISCUSSION

Results from this study reveal a role for the maternal microbiome in supporting placental growth and vascular development during pregnancy. We find that depletion of the maternal microbiome by oral treatment with ABX impairs placental development, which is prevented by supplementation with SCFAs derived from microbial fermentation of complex dietary carbohydrates, strongly suggesting that the maternal microbiome in particular in the gut is responsible. This distinction is notable in light of several studies reporting the existence of a microbiome within the placenta itself (*21*–*24*). Whether there are microbes native to the placental environment remains controversial, as additional evidence has suggested that microbial sequences detected in placental samples are due to laboratory contamination or reflect pathogenic entry into the human placenta, rather than a native placenta-associated microbiota (*25*, *26*). In addition, evidence supporting the presence of viable symbiotic microbes in the placenta, as opposed to microbial genetic material, captured by immune cells for example, is lacking. In mice, the existence of live microbes in the placenta that reach the fetus is repudiated by the ability to generate GF animals by cesarean hysterectomy shortly before parturition. Findings in this study in mice provide proof of principle that the maternal gut microbiome and gut microbial metabolites are required for supporting placental morphogenesis.

While all placentas and fetuses from each litter were assessed for weight, our experimental design did not account for any differences in fetal sex. This may be an issue in particular for assessments of feto-placental vasculature, placental transcriptomes, and placental imaging, where equal numbers of placentas were selected at random per litter for analysis. On the basis data provided by Ishikawa *et al.* (*27*), E14.5 placentas from XY fetuses were approximately 10% heavier than those from XX fetuses. In contrast, we observe that maternal microbiome deficiency results in ~15% decrease in placental weight, maternal PR results in 13% decrease in placental weight, and SCFA supplementation increases placental weight by 10%. Hence, we believe it is unlikely that differences in fetal sex can fully account for the phenotypes we observe in response to maternal microbiome modulation, maternal PR, and SCFA supplementation.

We find that maternal supplementation with SCFAs increases placental size and feto-placental vasculature across two mouse models, maternal microbiome-deficiency, and PR, which is not seen with maternal supplementation with a consortium of other key metabolites that are modulated by the maternal microbiome. While it is well established that GF and ABX mice exhibit low SCFA levels, our data indicate no difference in SCFA levels between dams fed a protein-restricted diet and those fed a CD. Hence, while it is possible that deficient SCFAs may contribute to placental impairments in microbiota-deficient dams, PR itself likely does not act through the same mechanisms to disrupt placental physiology. This further suggests that SCFA supplementation is effective for preventing PR-induced placental abnormalities, even when their etiopathogenesis likely occurs via a mechanism that does not involve SCFA deficiency. Consistent with our findings in a mouse model of maternal PR, maternal consumption of high-fat diet during pregnancy resulted in placental hypoxia and alterations in placental blood vessel structure, which were correlated with reductions in SCFA-producing bacteria in the maternal gut microbiota. Maternal supplementation with the SCFA butyrate prevented the placental impairments (*28*), supporting a role for SCFAs in promoting placental development.

While the mechanisms underlying the influences of SCFAs on the placenta remain unknown, SCFA concentrations increase with pregnancy (*29*) and reach fetal circulation via transplacental transport through monocarboxylate transporters (*30*). SCFA receptors are known to be expressed by uteroplacental tissues across mammals (*31*), suggesting the potential for direct receptor-mediated signaling of SCFAs to placental cells. Consistent with this, we show that select SCFAs increase branching tube formation by HUVEC cells through the cognate receptors FFAR2 and FFAR3, which may explain the ability of maternal SCFA supplementation during pregnancy to increase feto-placental vasculature in vivo. Aligning with this possibility, maternal SCFA supplementation increased butyrate and propionate levels in the fetal blood within 1 hour, suggesting entry of SCFAs into the placenta and transfer across feto-placental endothelial cells. More definitive experiments are necessary to assess whether the ability of maternal SCFA supplementation to promote feto-placental vascular development is mediated by direct receptor-mediated signaling of placental endothelial cells. Our study did not consider contributions of specific dietary fibers to SCFA production, which would extend the importance of key nutrients that promote feto-placental health. Both the control and low-protein diets used for the maternal PR experiments were formulated with cellulose as the main carbohydrate source because, as an insoluble fiber, it is largely indigestible to the host and poorly fermented by the mouse and human gut microbiome. This makes it more likely that effects seen in response to the PR diet are due to true PR rather than compensatory increases in fermentable carbohydrate. However, given that both PR diet and CD are formulated with cellulose at the expense of soluble fibers, it is likely that both result in equivalent decreases in baseline SCFAs in mice as compared to standard chow diets. Hence, it is possible that the beneficial effects of SCFA supplementation that we observe are context specific, dependent on the low soluble fiber composition of the diets used. Given that high-fiber diets have shown similar phenotypic rescue as SCFA supplementation in mice (*14*), our findings support the need to evaluate how varied dietary fibers affect feto-placental health.

Beyond interactions with the placenta, the maternal microbiota and SCFAs have been reported to support the development of fetal intestinal, pancreatic, and neural tissues in mice (*14*). In this study, we observed that fetuses from ABX dams, but not GF dams, were significantly smaller on E14.5 to 18.5 compared to SPF controls. Several studies have reported that the physiological outcomes of ABX treatment do not completely phenocopy those seen with GF rearing (*32*–*36*). Despite similar reductions in thalamocortical axon development in both GF and ABX dams, we previously found that only fetal brains from ABX dams were significantly reduced relative to controls (*2*). The differences seen with GF rearing could be due to host physiological alterations in response to the persistent absence of the microbiome during development, over the entire life span, and across several generations of GF breeding. This is in contrast to the acute and incomplete depletion of the microbiome by ABX treatment during adulthood. In this context, it is plausible that compensatory physiological adaptations to transgenerational GF rearing may explain the lack of phenotype in fetuses from GF dams compared to ABX dams. In addition, our present study demonstrates that absorbable ABX treatment (Abs) does not significantly impair placental weight but partially phenocopies reduced fetal weight observed in ABX and non-Abs litters, respectively. This implies that there are additional determinants of fetal weight that are distinct from those that determine placental weight in our study.

Reduced concentrations of the SCFA acetate have been reported in women with preeclampsia, and perinatal acetate supplementation is sufficient to promote fetal thymic CD4^+^ T cell and regulatory T cell development in GF mice (*37*). Whether effects of SCFAs on placental structure and function may contribute to the reported influences of SCFAs on fetal development requires further experimental investigation. However, placental insufficiencies, including vascular impairments, have long been linked to adverse outcomes in developing offspring and increased risk for chronic disease during adulthood (*38*). Placental vascular deficits are associated with reduced fetal weight and length, as well as reduced body mass, elevated systolic blood pressure, and reduced left ventricular mass during childhood (*39*). Consistent with this, reduced placental size is correlated with increased incidence of hypertension during adulthood (*38*). Moreover, placental vascular deficiencies characteristic of hypertensive disorders of pregnancy, and preeclampsia in particular, are associated with increased risk for myriad diseases in adulthood, including cardiovascular disease, kidney disease, and cognitive impairment (*40*). Fetal sex differentially affects placental and fetal growth (*27*), although our experimental design did not account for fetal sex in favor of unbiased quantifications based on litter averages. However, findings from our study reveal that metabolic functions contributed by the maternal gut microbiome during pregnancy are integral to supporting placental growth and vascularization in mice. Advancing our understanding of how the maternal gut microbiome affects placental structure and function may inform new approaches to promote maternal and fetal health and to decrease risk for chronic diseases.

## MATERIALS AND METHODS

### Experimental design

The objective of this study was to identify contributions of the maternal gut microbiome to placental development using gnotobiotic mouse models. In addition to characterization of placental phenotypes following microbiota depletion, we identified differentially regulated metabolites and evaluated whether these molecules modify placental deficiencies using in vivo and in vitro strategies.

### Mice

C57Bl/6J mice were purchased from Jackson Laboratories and either reared as SPF or rederived as GF and bred in flexible film isolators at the University of California, Los Angeles (UCLA) Center for Health Sciences barrier facility. Animals were maintained on a 12-hour light-dark cycle in a temperature-controlled environment, with autoclaved bedding. Sterile water and autoclaved standard chow (Lab Diet 5010) were provided ad libitum. For all experiments, pregnant dams were euthanized by cervical dislocation to preclude the effects of CO_2_ exposure on maternal and fetal physiology. All experiments were performed in accordance with the National Institutes of Health Guide for the Care and Use of Laboratory Animals using protocols (2015-077-01) approved by the Institutional Animal Care and Use Committee at UCLA.

#### Sample size determination

Eight-week-old mice were randomly assigned to experimental groups, which included age- and sex-matched cohorts of males and females for timed mating. To account for maternal microbiome status as the primary experimental variable in all animal experiments, biological sample sizes reflect the number of independent dams, and only virgin females were used for breeding. Characterization of placental and fetal phenotypes included at least two randomly selected conceptuses per litter for each individual dam, where litter averages represent biological “*n*.” For placental and fetal weight statistics, litter averages are presented in the main figures of the manuscript, and accompanying individual conceptus data are included either in main figures or in supplementary figures.

#### ABX treatment and conventionalization

For broad-spectrum ABX treatment, 8-week-old SPF mice were gavaged twice daily (08:00 and 17:00) for 1 week with a cocktail of neomycin (100 mg/kg), metronidazole (100 mg/kg), and vancomycin (50 mg/kg), based on methods previously described to mimic GF status (*41*). Ampicillin (1 mg/ml) was provided ad libitum in drinking water. Breeders were then paired and time mated. E0.5 was determined by observation of the copulation plug using a sterile vaginal probe. Dams were then separated, individually housed, and maintained on sterile drinking water supplemented with ampicillin (1 mg/ml), neomycin (1 mg/ml), and vancomycin (0.5 mg/ml) until E14.5 to preclude any stress of oral gavage in pregnant dams.

For selective ABX treatment, 8-week-old SPF mice were randomly selected for treatment with either ABX that are absorbed into host circulation (Abs) or with ABX that are not absorbed into host circulation (non-Abs). Abs mice were gavaged twice daily (08:00 and 17:00) for 1 week with metronidazole (100 mg/kg), and ampicillin (1 mg/ml) was provided ad libitum in drinking water. Non-Abs mice were gavaged twice daily (08:00 and 17:00) for 1 week with neomycin (100 mg/kg) and vancomycin (50 mg/kg) and then maintained on sterile drinking water with neomycin (1 mg/ml) and vancomycin (0.5 mg/ml). Breeders were paired and time mated, and dams were separated on E0.5 as described above.

#### Conventionalization of GF mice

To generate GF conventionalized controls, mice born and reared GF were colonized with a conventional mouse microbiota during adulthood. SPF donor fecal pellets were freshly homogenized at 100 mg/ml in prereduced sterile phosphate-buffered saline (PBS), and 200 μl was gavaged into each 8-week-old female GF recipients once per week for 2 weeks. Bedding from the SPF donor home cage was also added to the GF CONV recipient cage to maximize conventionalization.

#### Maternal protein-restriction

Eight-week-old mice previously fed standard chow (Lab Diet 5010) were randomly selected to receive either CD with 20.3% protein by weight, 61.3% carbohydrate by weight, and 5.5% fat by weight (TD.91352, Envigo) or protein-restricted diet with 6.1% protein by weight, 75.6% carbohydrate by weight, and 5.5% fat by weight (TD.90016, Envigo). Differences in carbohydrates resulted from amounts of sucrose and cellulose, and both diets used were isocaloric by weight (3.8 kcal/g) and matched for calcium (0.7%) and phosphorous (0.54%). Both male and female mice acclimated to the respective diets for a 2-week period and were then paired for breeding within the respective dietary treatment groups. Upon observation of the copulation plug at E0.5, females were individually housed and maintained on the respective diet for the duration of gestation.

### 16*S* rRNA gene sequencing

Bacterial genomic DNA was isolated from mouse fecal samples following the standard protocol from the DNeasy PowerSoil Kit (Qiagen). Sequencing libraries were generated according to methods adapted from Caporaso *et al.* (*42*), amplifying the V4 regions of the 16*S* ribosomal RNA (rRNA) gene by polymerase chain reaction (PCR) using individually barcoded universal primers and 30 ng of the extracted genomic DNA. Each PCR reaction was performed in triplicate and pooled following amplification. The final PCR product was purified using the Qiaquick PCR purification kit (Qiagen). A total of 250 ng of purified, PCR product from each individually barcoded sample was pooled and sequenced by Laragen Inc. using the Illumina MiSeq platform and 2 × 250 bp reagent kit for paired-end sequencing. All analyses were performed using QIIME2 (*43*), including Deblur for quality control (*44*), taxonomy assignment, alpha-rarefaction, and beta-diversity analyses. A total of 29,152 reads were analyzed per sample. Operational taxonomic units were assigned on the basis of 99% sequence similarity compared to the SILVA 132 database. Alpha-rarefaction curves and beta-diversity principal coordinates analysis plots were generated using Prism software (GraphPad) and QIIME2 View, respectively, and taxa bar plots were generated using Microsoft Excel.

### Immunofluorescence staining

E14.5 placentas were fixed in 4% paraformaldehyde for 24 hours at 4°C, cryoprotected in 30% sucrose in PBS for 24 hours at 4°C, and sectioned at 12 μm using a Leica CM1950 cryostat. Sections were blocked with 10% goat serum for 1 hour at room temperature. Primary antibodies were diluted in 10% goat serum and incubated for 18 hours at 4°C with laminin rabbit anti-mouse antibody (1:250, Sigma-Aldrich, L9393) or CD31 rat anti-mouse antibody (1:100, Thermo Fisher Scientific, 14-0311-82), respectively. Sections were then incubated for 1 hour at room temperature in their corresponding goat anti-rabbit antibodies conjugated to Alexa Fluor 568 (1:1000, Thermo Fisher Scientific) and 4′,6-diamidino-2-phenylindole. Images were acquired using the Zeiss Axio Examiner LSM 780 confocal microscope with 405 nm (0.2% laser line attenuator transmission, 575 master gain, 0 digital offset, 1.0 digital gain) and 561 nm (2.0% laser line attenuator transmission, 605 master gain, −2.0 digital offset, 1.0 digital gain) lasers. One to two placental sections were scanned for each sample using Zen Black 2012 software, 20× objective at 1.5× zoom with 5 × 1 μm interval *z* slices, and three individual tracks for each fluorescent dye. Image acquisition settings included as follows: scan mode set at frame, frame size set at 1024 × 1024, scan speed set at 7, averaging at 2 by line and mean, and bit depth set at 8 bit. Pinhole was set to 1 AU. Images were stitched with a 10% overlap, and the complete range of *z* series was compressed using Zen Blue 2021 software.

#### Image analysis

All acquired images were analyzed using the same procedures by a researcher blinded to the experimental group of each sample. Images were imported into Fiji and calibrated using a set scale. The perimeter of the labyrinth area was manually traced, and labyrinth area was calculated by oversaturating all tissue using brightness and contrast settings from 0 minimum to 8 maximum, where areas of no signal were defined as void spaces. To quantify integrated density of laminin, brightness and contrast settings were adjusted to eliminate background autofluorescence. Raw integrated density values for each sample were then normalized to the total labyrinth area for each sample.

### Micro–computed tomography

Whole E14.5 placentas were serially dehydrated, from 30 to 50 to 70% ethanol and incubated in 4% (w/v) phosphotungstic acid diluted in 70% ethanol for 4 days at 4°C. Tissues were scanned at 60 kVp/150 μA with a 1-mm Al filter at 8 μm resolution using a benchtop μCT scanner (SkyScan 1275, Bruker). Reconstructions of two-dimensional images were generated using dynamic range adjustment and gaussian smoothing, a ring artifact reduction of 10, and a defect pixel mask of 8%. Volumes of interest for fetal labyrinth volume measurements were selected manually by a blinded researcher, using labyrinth density and tissue morphology to distinguish the labyrinth from the overlying junctional zone and decidua. Whole placental volumes were reconstructed and measured using CTAn and CTVol software (Bruker Corporation), using a threshold range of 88 (minimum) to 255 (maximum). Whole-placenta representative images were created using Dataviewer software (Bruker Corporation), with heatmaps reflecting Houndsfield units as a metric of radio density. Quantification of whole volume of E14.5 fetuses was conducted using μCT images that were used for brain volume quantification in (*2*).

#### Placental arterial vascular casts

Feto-placental arterial vascular casts were generated adapting previously described methods (*45*). Briefly, dams were euthanized on E14.5 by cervical dislocation, and uterine horns were dissected immediately and placed in ice-cold PBS. Conceptuses were individually removed, placed on a heating pad set at 37°C, and continuously flushed with warmed (37°C) PBS to restore fetal heartbeat. The fetal umbilical artery was identified following observation of pulsing blood flow, 4% PFA in PBS was dropped onto the artery for partial vasodilation, and a micro-incision was made on the artery. A hand-pulled glass pipette affixed to tubing was inserted into the arterial lumen, and silicone adhesive Kwik-Sil (World Precision Instruments) was used to cover the incision site and adhere the pipette in place. Once the silicone adhesive was set, a small incision was made on the umbilical vein to serve as an outflow for perfusions. To flush the feto-placental vasculature and preclude vasoconstriction, a warmed (37°C) 1:1 solution of heparinized (100 IU/ml) PBS and 10% lidocaine was perfused through the placenta until the perfusate was free of blood. Without introducing air bubbles, a tubing line with ice-cold MICROFIL silicone rubber injection compound (Flow Tech Inc.) was perfused through the umbilical artery until resistance was met to maintain pressure. Both umbilical vessels were immediately sutured and filled placentas were placed in room temperature PBS for at least 30 min for casting compound to polymerize. Samples were then serially dehydrated in ethanol, from 30, 50, into 70% ethanol at 4°C to reduce background electron density from placental tissue. Perfused placental casts were imaged by μCT, as described above. Feto-placental arterial volume and surface area were determined following three-dimensional reconstruction using CTAn and CTVol software (Bruker Corporation). Notably, the limit of resolution of μCT (1 voxel = 11 μm) prevents us from detecting the smaller, dense labyrinth microvasculature (*46*, *47*), which likely accounts for the lower density of vessels observed by this method as compared to the more resolved staining and confocal imaging. We therefore performed both vascular perfusions and histological analyses as complementary approaches to circumvent this issue.

### Metabolomics

At E14.5, maternal blood was collected by cardiac puncture and fetal blood was collected by pooling trunk blood from multiple decapitated fetuses, where individual datapoints reflect pooled litter averages. Serum was separated using SST vacutainer tubes (Beckton Dickinson) and frozen at −80°C. Samples were prepared using the automated MicroLab STAR system (Hamilton Company) and analyzed on gas chromatography–mass spectrometry (GC-MS), liquid chromatography–MS (LC-MS), and LC-MS/MS platforms by Metabolon Inc. Organic aqueous solvents were used to perform serial extractions for protein fractions, concentrated using a TurboVap system (Zymark) and vacuum dried. For LC-MS and LC-MS/MS, samples were reconstituted in acidic or basic LC-compatible solvents containing >11 injection standards and run on a Waters ACQUITY UPLC and Thermo-Finnigan LTQ mass spectrometer, with a linear ion-trap frontend and a Fourier transform ion cyclotron resonance mass spectrometer back-end. For GC-MS, samples were derivatized under dried nitrogen using bistrimethyl-silyl-trifluoroacetamide and analyzed on a Thermo-Finnigan Trace DSQ fast-scanning single-quadrupole mass spectrometer using electron impact ionization. Chemical entities were identified by comparison to metabolomic library entries of purified standards. Following log transformation and imputation with minimum observed values for each compound, data were analyzed using one-way analysis of variance (ANOVA) to test for group effects. *P* and *q* values were calculated on the basis of two-way ANOVA contrasts. Principal components analysis was used to visualize variance distributions. Supervised random forest analysis, a machine learning algorithm to classify and regress input data using multiple decision trees, was conducted to identify metabolomics prediction accuracies. All reference data are listed in table S1. SCFAs were not detected using these methods because they require different extraction methods that account for their partial hydrophilicity. We therefore complemented this untargeted metabolomic profiling with targeted SCFA quantification, as described further below.

#### Candidate metabolite selection for supplementation experiments

Physiologically relevant metabolite concentrations were determined on the basis of reported murine serum concentrations from the mouse multiple tissue metabolomic database, human serum from the human metabolome database, and existing literature. All reference literatures are listed in table S2. Metabolites of interest were selected on the basis of random forest analysis identifying the top 30 distinguishing features of either fetal serum or maternal serum [previously published in (*2*)], respectively, and selection criteria are listed in table S2. Metabolites not reasonably available commercially were not considered for experimentation. Last, all compounds with known teratogenic effects were eliminated from our in vivo supplementation to avoid confounding effects on gestational development.

### In vivo metabolite supplementation

To test the effects of the candidate microbiome-dependent fetal metabolites, the Metab cocktail or vehicle control was administered intraperitoneally once daily from E0.5 to E14.5 to minimize stress to pregnant dams. Metabolite dosages were calculated on the basis of fetal serum metabolomic data and physiologically relevant metabolite concentrations reported in literature, to reflect the daily levels needed to match those observed in SPF fetal serum (table S2). Metabolite concentrations were calculated on the basis of physiological levels in mouse or human blood (table S2), total blood volume of pregnant mouse dams [approximately 58.5 ml/kg (*48*)], and relative reductions observed in fetal sera of ABX fetuses compared to SPF fetuses (table S2). The metabolite stock solution consisted of: 29.64 μM imidazole propionate, 714.096 μM *N*,*N*,*N*-trimethyl-5-aminovalerate, 13.452 μM 4-hydroxyphenylacetate, 316.008 μM phenol sulfate, 428.868 μM indolepropionate, 360.24 μM indoxyl glucuronide, 323.76 μM *N*-methylproline, 595.080 μM phenylacetylglycine, 957.6 μM trimethylamine *N*-oxide, 118.56 μM taurodeoxycholate, 160.8768 μM biotin, 893.76 μM hippurate, 42.864 μM 2-(4-hydroxyphenyl)propionate, 222.072 μM cinnamoylglycine, 273.6 nM equol glucuronide, 433.2 μM 2-aminophenol sulfate, 1.55952 mM 3-indoxyl sulfate, and 268.128 μM p-cresol sulfate in 0.1 M PBS. The stock solution was then diluted 1:100 in sterile 0.1 M PBS, and 200 μl of the working solution was injected intraperitoneally into E0.5 ABX dams once a day daily for 14 days. For SCFA treatment, sodium propionate (25 mM), sodium butyrate (40 mM), and sodium acetate (67.5 mM) were supplemented to drinking water of pregnant ABX dams from E0 to E14.5. These concentrations were determined on the basis of existing studies demonstrating that these concentrations were able to sufficiently penetrate host tissues distal from the gut and restore circulating physiological concentrations (*12*). SCFA-supplemented drinking water and sodium-matched control drinking water were sterile filtered and made fresh every 4 days. To assess placental phenotypes, dams treated with metabolites were euthanized on E14.5, and placentas were harvested and processed as described in sections above.

#### SCFA quantification from E14.5 tissues

To assess whether intragastric SCFA administration in ABX dams increases tissue-specific concentrations of acetate, propionate, and butyrate, dams treated with SCFA-supplemented or sodium-matched control drinking water, respectively, were fasted overnight to normalize circulating concentrations. SCFAs have previously been demonstrated to cross the transplacental barrier to detectable concentrations in fetal tissues (*14*). Fasted E14.5 dams were orally gavaged with 200 μl of either SCFA-stock or sodium-matched solution, respectively, and euthanized 1 hour later for tissue collection. Maternal blood was collected by cardiac puncture and fetal blood was collected by pooling trunk blood from decapitated fetuses. Maternal serum was separated using SST vacutainer tubes (Beckton Dickinson) and frozen at −80°C. Three randomly selected placentas were collected from each litter and frozen at −80°C. A total of 150 to 250 mg of tissue samples were homogenized in 150 μl of PBS (Bead Mill 24, Thermo Fisher Scientific), vortexed, and sonicated in ice water for 10 min. Tissue lysate was then centrifuged at 14,000*g* for 10 min at 4°C and the supernatant was collected. Fifty microliters of serum/whole blood or above tissue lysate supernatant was mixed well with 50 μl of isopropyl alcohol with isotope-labeled internal standards (acetic-d3, propionic-d5, and butyric-d7, 5 μg/ml each), precipitated at −20°C overnight, and centrifuged at 14,000*g* for 10 min at 4°C. Forty microliters of supernatant was transferred to a clean tube and reacted with 20 μl of 200 mM 3-nitrophenylhydrazine hydrochloride in 50% aqueous acetonitrile and 20 μl of 120 mM *N*-(3-dimethylaminopropyl)-*N*′-ethylcarbodiimide hydrochloride in 50% aqueous acetonitrile with 6% pyridine at 40°C for 30 min. The reaction was diluted with 80 μl of 0.5% formic/H_2_O by vortex, centrifuge, and ready for LC-MS. The LC-MS analysis was performed on an Agilent Zorbax SB-C18 2.1X150 mm at a column temperature of 50°C. Eluent A consisted of 0.1% formic acid acetonitrile. Eluent B consisted of 0.1% formic acid in water. Gradient elution was performed with the following: 5% A to 25% A in 30 min and 25% A to 40% A in 10 min, with flow rate 0.2 ml/min. Analyses were performed using the TSQ Quantum (Thermo-Finnigan) LC–electrospray ionization–MS/MS system at negative mode. Quantification was achieved by using Xcalibur data system. Acetate: mass/charge ration (*m*/*z*) 194.1/137.1 (197.1/137.1for acetic d3); propionate: *m*/*z* 208.1/137.1(213.1/137.1 for propionic d5); and butyrate: *m*/*z* 222.1/137.1(229.1/137.1 for butyric d7). Calibration curves were built by fitting the analyte concentrations versus the peak area ratios of the analyte to isotope-labeled internal standards. The peak area ratios of target analyte to isotope-labeled internal standards in the samples were used to calculate the concentrations.

### Doppler ultrasound analyses

Methods adopted for Doppler ultrasound analyses of placental labyrinth and umbilical vessels were performed as previously described (*49*). Briefly, E14.5 dams were initially anesthetized with isoflurane at 3.5%, transferred to a heated stage to maintain 37°C body temperature in a dorsal position, and remained anesthetized affixed to an anesthesia nose tube with 1.5% isoflurane and O_2_. Electrode gel was applied to each electrode, and dams were secured to the heated stage by taping each limb over its corresponding electrode with surgical tape. Body temperature, respiratory rate, and maternal heart beat (450 to 550 beats/min) were monitored for the duration of the experiment. Depilatory cream was applied to the abdomen, allowed to rest for 3 min, and hair was wiped off with a water-soaked compress. Prewarmed ultrasound gel was applied to the depilated skin. Ultrasound imaging and pulse-wave Doppler recordings (angle of incidence parallel to blood flow) were captured with a Vevo 3100 (Fujifilm, VisualSonics), and at least three random conceptuses were imaged per litter. Doppler recordings were analyzed using Vevo Lab software (Fujifilm, VisualSonics). Each conceptus had two to three recordings from both placental labyrinth and umbilical vessels, respectively, which were averaged as technical replicates for that conceptus. Values from all conceptuses from the same litter were then averaged to create a single measured value per dam.

### HUVEC tube formation assay

First-generation HUVECs (Thermo Fisher Scientific, C0035C) were passaged to third or fourth generations in Medium 200 (Thermo Fisher Scientific) with large vessel endothelial supplement (LVES; Thermo Fisher Scientific) and penicillin (10 U/ml)/streptomycin (10 μg/ml; Sigma-Aldrich) at 37°C, 5% CO_2_. Branching tube formation assays were performed using tissue culture–treated μslides (Ibidi). Briefly, 10 μl of ice-cold Geltrex LVES-free Reduced Growth Factor Basement Membrane Matrix (Thermo Fisher Scientific) was added using prechilled pipettes to each well of a chilled μslide, and μslides were transferred to 37°C for 30 min for matrix polymerization following confirmation of equal matrix distribution across all wells. Third- or fourth-generation HUVECs were resuspended in LVES-free Medium 200 (minimal endothelial cell media) with penicillin (10 U/ml)/streptomycin (10 μg/ml; Sigma-Aldrich), and then resuspended with metabolite-supplemented media at the concentrations indicated in table S3 at 2 × 10^5^ cells/ml, and 50 μl of cell suspension was added to each well. HUVECs in LVES-free Medium 200 were used as a negative control, and Medium 200 with 2% fetal bovine serum (FBS) was used as a positive control. All conditions were performed in triplicate for each experiment, and each experiment was repeated three times. μslides were incubated at 37°C, 5% CO_2_ for 12 hours and imaged using a Leica DMi8 microscope. Image analysis and quantification was performed using Fiji (*50*) and the Angiogenesis analyzer plugin (*51*), and total branching distance values were averaged first for technical replicate by experiment, and then by biological replicate across multiple experiments, which is displayed in final figures.

### CRISPR-Cas9 ribonucleoprotein genomic editing in HUVECs

Guide RNA design, CRISPR-Cas9 ribonucleoprotein (cRNP) complex formation, and electroporation were adopted from methodology designed for genomic editing in primary innate immune cells (*52*). Guide RNA sequences were derived from recent whole-genome–based CRISPR-Cas9 KO libraries (*53*). We selected the top six single guide RNA (sgRNA) sequences for *FFAR2* and top four sgRNA sequences for *FFAR3* using Chopchop (*54*) based on predicted editing efficiency and number of off-target mismatches to test in HUVECs. HUVECs (1 × 10^6^) in T buffer (Thermo Fisher Scientific) were combined with complexed cRNP for each reaction, and electroporated using the Neon Transfection System (Thermo Fisher Scientific) at pulse code 1800 V 20 ms × 1 pulse. Immediately following electroporation, cells were resuspended in HUVEC media to dilute T buffer and incubated at 37°C for 90 min. Cells were then centrifuged and resuspended in HUVEC media with 10% dimethyl sulfoxide and stored in a liquid-nitrogen cell freezer for following knockout efficiency and in vitro HUVEC tube formation assays. To assess knockout efficiency, guide target sequences were amplified by PCR from genomic DNA isolated from transfected HUVECs, sequenced, and evaluated for indel % and knockout score using the SYNTHEGO ICE platform. The final sgRNA used for *FFAR2* (guide sequence, GCTGCAATCACTCCATACAGAGG) had a 41% indel and 41 knockout score. The final sgRNA used for *FFAR3* (guide sequence, AAAGTCGGCTTGGAACCCGGAGG) had an 86% indel and 83 knockout score. To test the effects of SCFAs signaling with both free-fatty acid receptors, FFAR3KO HUVECs were treated with the FFAR2 inhibitor GLPG 0974 (Tocris) at 10 μM.

### Statistical analysis

Statistical analysis was performed using Prism software (GraphPad). Data were assessed for normal distribution and plotted in the figures as mean ± SEM. For each figure, unless otherwise specified, *n* reflects the number of independent maternal biological replicates. Whole litters were only excluded if the total number of viable conceptuses was less than 4, and otherwise, no conceptuses were excluded from any litters represented. Differences among 2 or more groups with only one variable were assessed using one-way ANOVA with Tukey post hoc test. Individual placental and fetal weights, respectively, were analyzed by one-way nested ANOVA with Tukey post hoc test. Taxonomic comparisons from 16*S* rRNA gene sequencing analysis were analyzed by Kruskal-Wallis test with Tukey’s post hoc test. Differences between only 2 groups with one variable were assess using unpaired two-tailed *t* test. Two-way ANOVA with Tukey post hoc test was used for comparison of 2 or more groups with two variables. For all figures, significant differences emerging from the above tests are indicated by *P < 0.05, **P < 0.01, ***P < 0.001, and ****P < 0.0001, and notable nonsignificant differences are indicated by “n.s.”.
