# Supplementary material for: The maternal microbiome promotes placental development in mice
Source: Sci Adv. 2023 Oct 6;9(40):eadk1887. doi: 10.1126/sciadv.adk1887 (PMC10558122; doi:10.1126/sciadv.adk1887)
Supplement: Supplementary file 1 — Fig. S1 to S14 [file sciadv.adk1887_sm.pdf]

Science Advances
AAAS

Supplementary Materials for

The maternal microbiome promotes placental development in mice

Geoffrey N. Pronovost *et al.*

Corresponding author: Geoffrey N. Pronovost, geoffpronovost@gmail.com,

Elaine Y. Hsiao, ehsiao@g.ucla.edu

Sci. Adv. **9**, eadk1887 (2023)
DOI: 10.1126/sciadv.adk1887

The PDF file includes:

Fig. S1 to S14

Other Supplementary Material for this manuscript includes the following:

Tables S1 to S5
Data S1 to S18

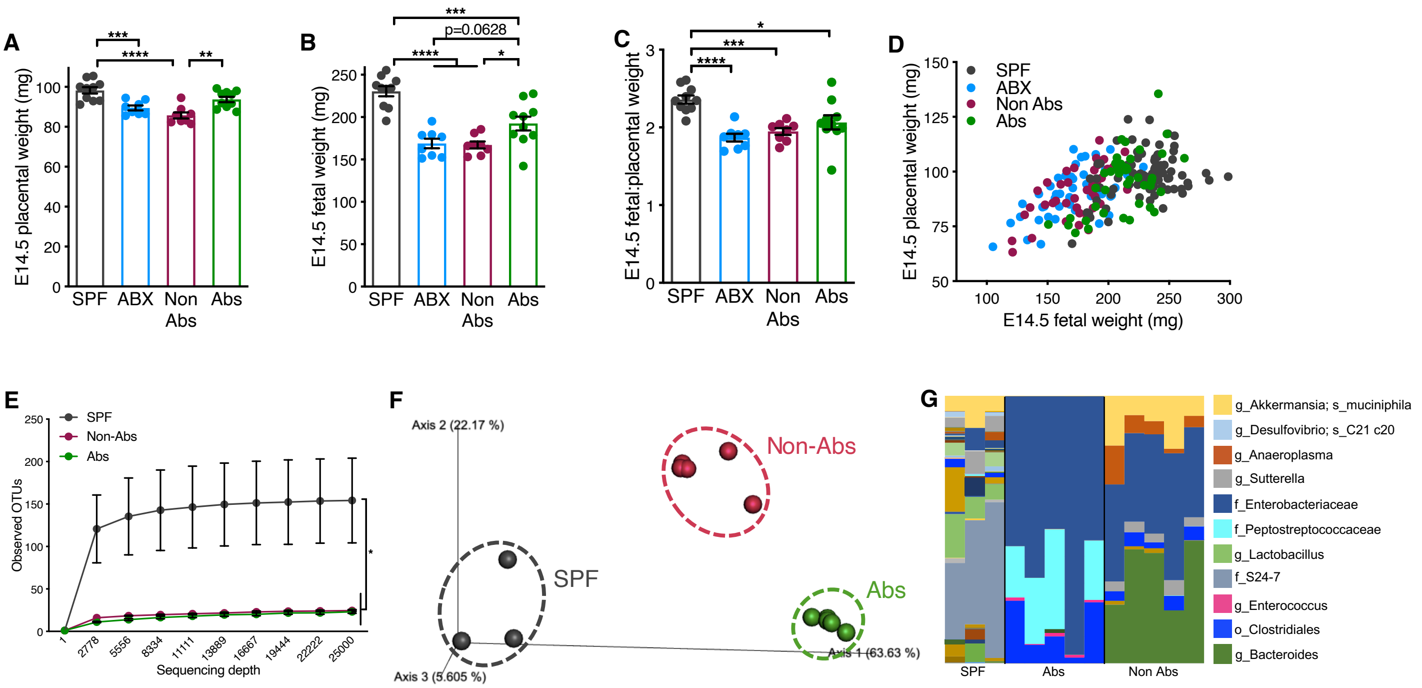

Fig. S1: Antibiotic-induced reductions in placental and fetal weight are not due to off-target effects of absorbable antibiotics. (A) E14.5 placental weights by litter average (SPF (n = 10; same as shown in Fig. 1), ABX (ampicillin, metronidazole, neomycin and vancomycin; n = 9), Non-absorbable antibiotics (Non-Abs; neomycin and vancomycin; n = 8) and absorbable antibiotics (Abs; ampicillin and metronidazole; n = 10). **(B)** E14.5 fetal weights by litter average from litters shown in Fig. S1A. **(C)** E14.5 fetal to placental weight ratios for litters shown in Fig. S1A-B. **(D)** Linear correlation of E14.5 placental to fetal weight ratios for all individual conceptuses (SPF (n = 80), ABX (n = 49), Non-Abs (n = 40) and Abs (n = 42)) from litters shown in Fig. S1A-C. **(E)** Alpha-rarefaction curves of 16S rRNA gene sequencing results from E14.5 maternal fecal samples collected from independent cages (SPF (n = 3), Non-Abs (n = 5) and Abs (n = 5)). **(F)** Weighted UniFrac analysis of 16S rRNA gene sequencing results from E14.5 maternal fecal samples shown in Fig. S1E. **(G)** Taxa bar plots for 16S rRNA gene sequencing results from E14.5 maternal fecal samples shown in Fig. S1E-F. Data represent mean ± SEM; statistics were performed with one-way ANOVA with Tukey *post hoc* test. $*P < 0.05$; $**P < 0.01$; $***P < 0.001$; $****P < 0.0001$.

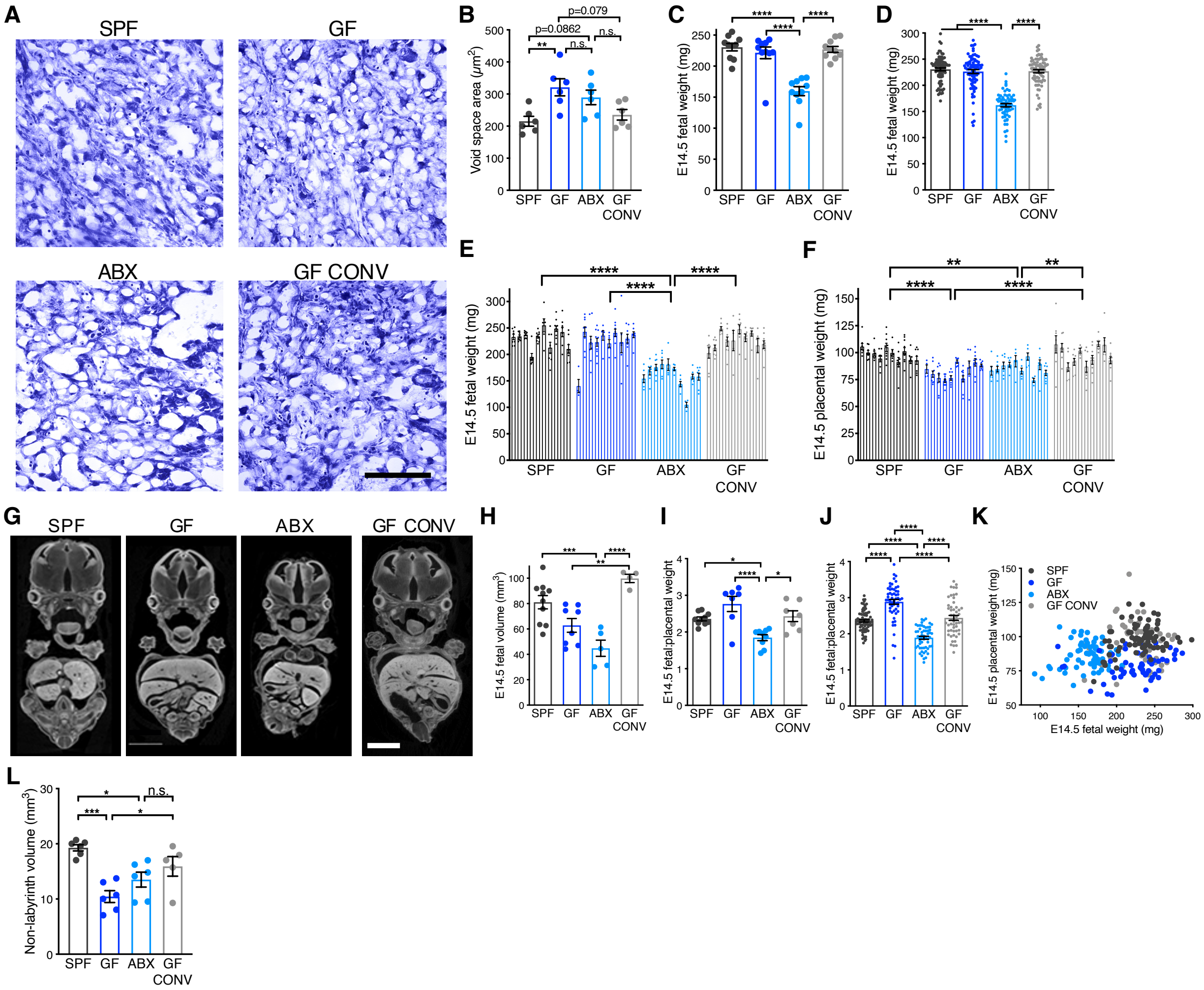
A
SPF
GF
ABX
GF CONV
B
Void space area (μm²)
p=0.079
p=0.0862
**
n.s.
n.s.
SPF GF ABX GF CONV
C
E14.5 fetal weight (mg)
****
****
****
SPF GF ABX GF CONV
D
E14.5 fetal weight (mg)
****
****
SPF GF ABX GF CONV
E
E14.5 fetal weight (mg)
****
****
****
SPF GF ABX GF CONV
F
E14.5 placental weight (mg)
**
**
****
****
SPF GF ABX GF CONV
G
SPF
GF
ABX
GF CONV
H
E14.5 fetal volume (mm³)
***
****
**
SPF GF ABX GF CONV
I
E14.5 fetal:placental weight
*
****
*
SPF GF ABX GF CONV
J
E14.5 fetal:placental weight
****
****
****
****
****
SPF GF ABX GF CONV
K
SPF
GF
ABX
GF CONV
E14.5 placental weight (mg)
E14.5 fetal weight (mg)
L
Non-labyrinth volume (mm³)
*
n.s.
***
*
SPF GF ABX GF CONV

Fig. S2: Maternal microbiome depletion differentially affects E14.5 fetal weight. (A) Representative images of thionin-stained E14.5 placental labyrinth tissues from SPF, GF, ABX and GF CONV litters (scale bar = 250 µm)**. (B)** Quantification of average placental labyrinth void space area from litters shown in Fig. S2A (SPF *(n* = 6), GF *(n* = 6), ABX *(n* = 6) and GF CONV *(n* = 6)). (**C)** E14.5 fetal weights by litter average (SPF (*n* = 10), GF (*n* = 10), ABX (*n* = 10) and GF CONV (*n* = 10). (**D)** E14.5 fetal weights for each individual from litters shown in Fig. S2C (SPF (*n* = 80), GF (*n* = 81), ABX (*n* = 65) and GF CONV (*n* = 77). **(E)** Individual E14.5 fetal weights used for 1-way nested statistical analysis from litters shown in Fig. S2C (SPF (*n* = 80), GF (*n* = 81), ABX (*n* = 65) and GF CONV (*n* = 77). **(F)** Individual E14.5 placental weights used for 1-way nested statistical analysis from litters shown in Fig. S2C (SPF (*n* = 80), GF (*n* = 81), ABX (*n* = 65) and GF CONV (*n* = 77). **(G)** Representative cross-sections of E14.5 whole fetal µCT reconstructions from SPF, GF, ABX and GF CONV litters (scale = 1mm). µCT images in this study are the same as those used for brain volume quantitation in Vuong et al, 2020. **(H)** Quantification of E14.5 whole fetal volumes from µCT reconstructions by litter average (SPF (*n* = 10), GF (*n* = 8), ABX (*n* = 5) and GF CONV (*n* = 4). **(I)** E14.5 fetal to placental weight ratio for litters shown in Fig. S2C (SPF (*n* = 10), GF (*n* = 10), ABX (*n* = 10) and GF CONV (*n* = 10). **(J)** E14.5 fetal to placental weight ratios for individual conceptuses from litters shown in Fig. S2G (SPF (*n* = 80), GF (*n* = 81), ABX (*n* = 65) and GF CONV (*n* = 77) litters. **(K)** Linear correlation of E14.5 placental to fetal weight ratios for individual conceptuses shown in Fig. S2H. **(L)** Quantification of E14.5 non-labyrinth placental volumes from µCT reconstructions by litter average (SPF (*n* = 6), GF (*n* = 6), ABX (*n* = 6) and GF CONV (*n* = 5)). Data represent mean ± SEM; statistics were performed with one-way ANOVA with Tukey *post hoc* test (litter averages) or with 1-way nested with Sidak multiple comparisons correction (individual conceptuses). $^{*}P < 0.05$; $^{**}P < 0.01$; $^{***}P < 0.001$; $^{****}P < 0.0001$.

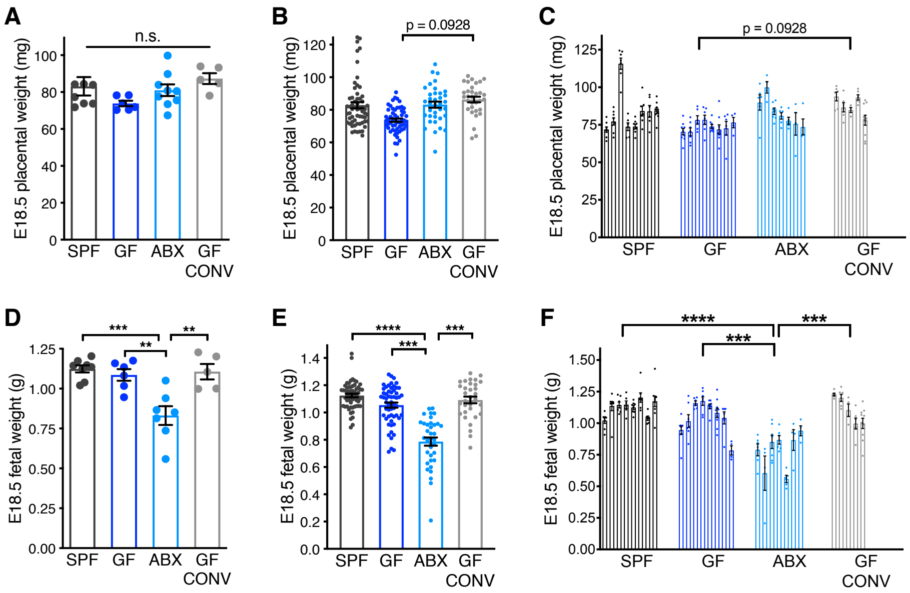

Fig. S3: Maternal microbiome depletion differentially affects E18.5 fetal weight. (A) E18.5 placental weights by litter average (SPF (*n* = 7), GF (*n* = 6), ABX (*n* = 9) and GF CONV (*n* = 5). **(B)** E18.5 placental weights for each individual from litters shown in Fig. S3A (SPF (*n* = 59), GF (*n* = 56), ABX (*n* = 36) and GF CONV (*n* = 32). **(C)** Individual E18.5 placental weights used for 1-way nested statistical analysis from litters shown in Fig. S3A (SPF (*n* = 59), GF (*n* = 56), ABX (*n* = 36) and GF CONV (*n* = 32). **(D)** E18.5 fetal weights by litter average from litters shown in S3A (SPF (*n* = 7), GF (*n* = 6), ABX (*n* = 9) and GF CONV (*n* = 5). **(E)** E18.5 fetal weights for each individual from litters shown in Fig. S3A (SPF (*n* = 59), GF (*n* = 56), ABX (*n* = 36) and GF CONV (*n* = 32). **(F)** Individual E18.5 fetal weights used for 1-way nested statistical analysis from litters shown in Fig. S3A (SPF (*n* = 59), GF (*n* = 56), ABX (*n* = 36) and GF CONV (*n* = 32). Data represent mean ± SEM; statistics were performed with one-way ANOVA with Tukey *post hoc* test (litter averages) or with 1-way nested with Sidak multiple comparisons correction (individual conceptuses). $**P < 0.01$; $***P < 0.001$; $****P < 0.0001$.

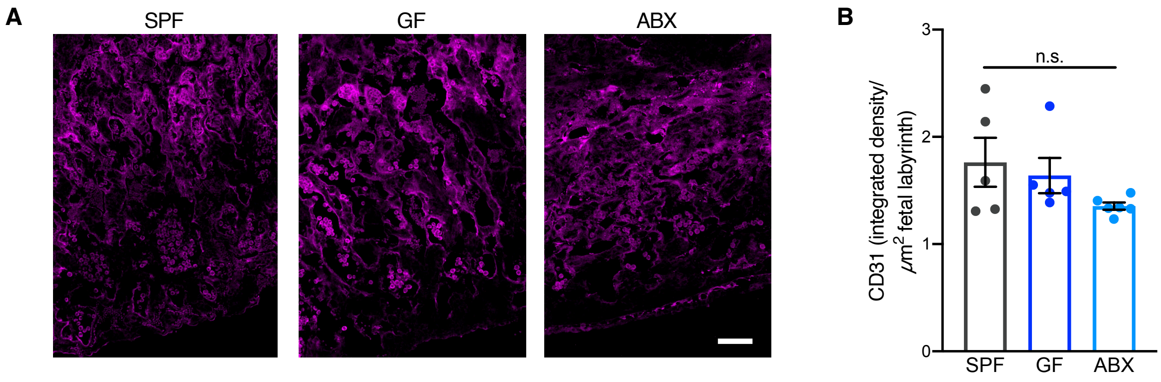

Fig. S4: E10.5 microvasculature density is not affected by maternal microbiota depletion. **(A)** Representative images of CD31-stained E10.5 placental labyrinth from SPF, GF and ABX (scale bar = 50 µm). **(B)** Quantification of raw integrated density of CD31 staining intensity, normalized to fetal labyrinth total area (SPF (n = 5), GF (n = 5) and ABX (n = 6)). Data represent mean ± SEM; statistics were performed with one-way ANOVA with Tukey *post hoc* test.

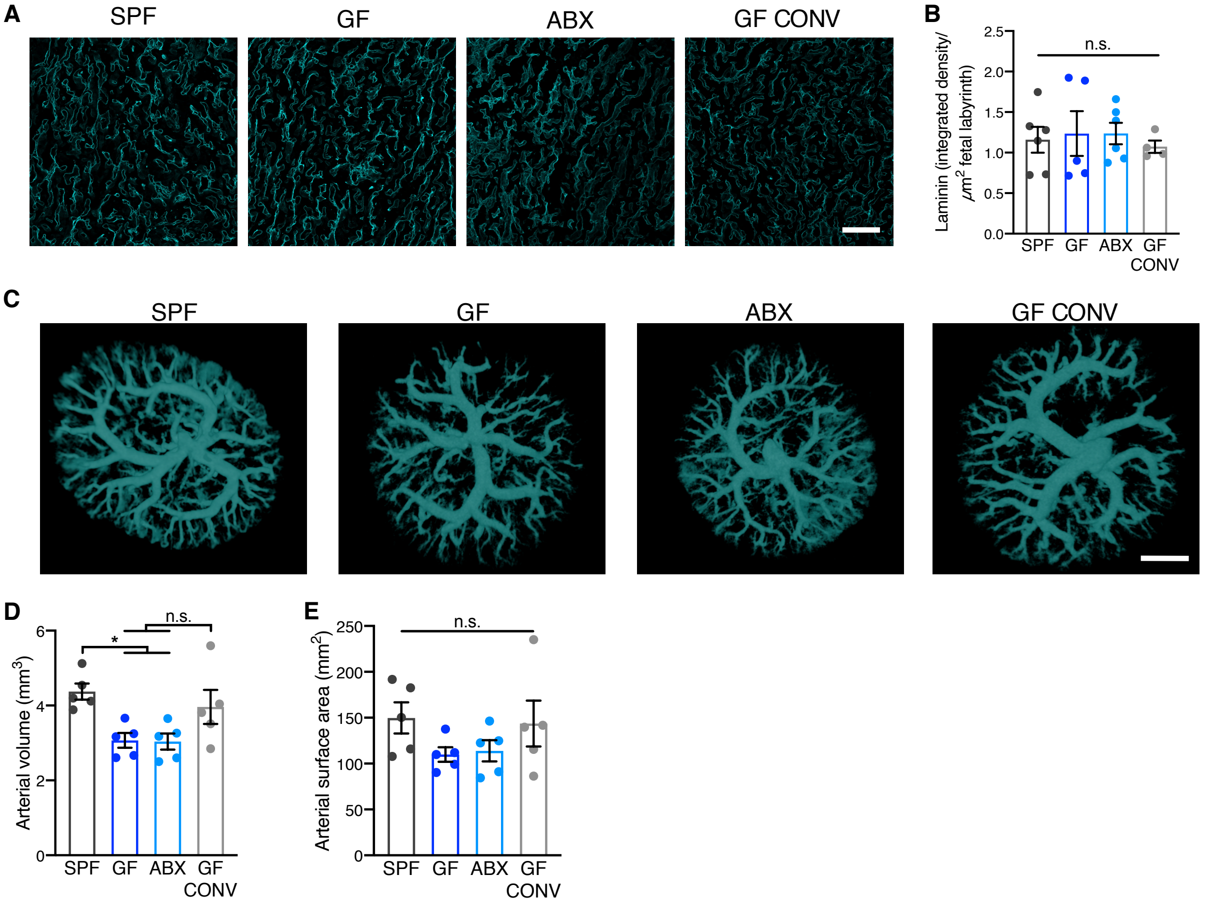

Fig. S5: The maternal microbiome promotes placental vascular development in the late-term placenta. (A) Representative images of laminin-stained E18.5 placental labyrinth from SPF, GF, ABX and GFCONV litters (scale bar = 100 µm). **(B)** Quantification of raw integrated density of laminin staining intensity, normalized to fetal labyrinth total area (SPF (n = 6), GF (n = 5), ABX (n = 5) and GF CONV (n = 4)). **(C)** Representative feto-placental arterial vascular reconstructions by µCT imaging of vascular casts from E18.5 SPF, GF, ABX and GF CONV litters; scale bar = 1 mm. **(D)** Quantification of E18.5 feto-placental arterial vascular volume (SPF (n = 5), GF (n = 5), ABX (n = 5) and GF CONV (n = 5)). **(E)** Quantification of E18.5 feto-placental arterial vascular surface area (SPF (n = 5), GF (n = 5), ABX (n = 5) and GF CONV (n = 5)). Data represent mean ± SEM; statistics were performed with one-way ANOVA with Tukey *post hoc* test. *$P < 0.05$.

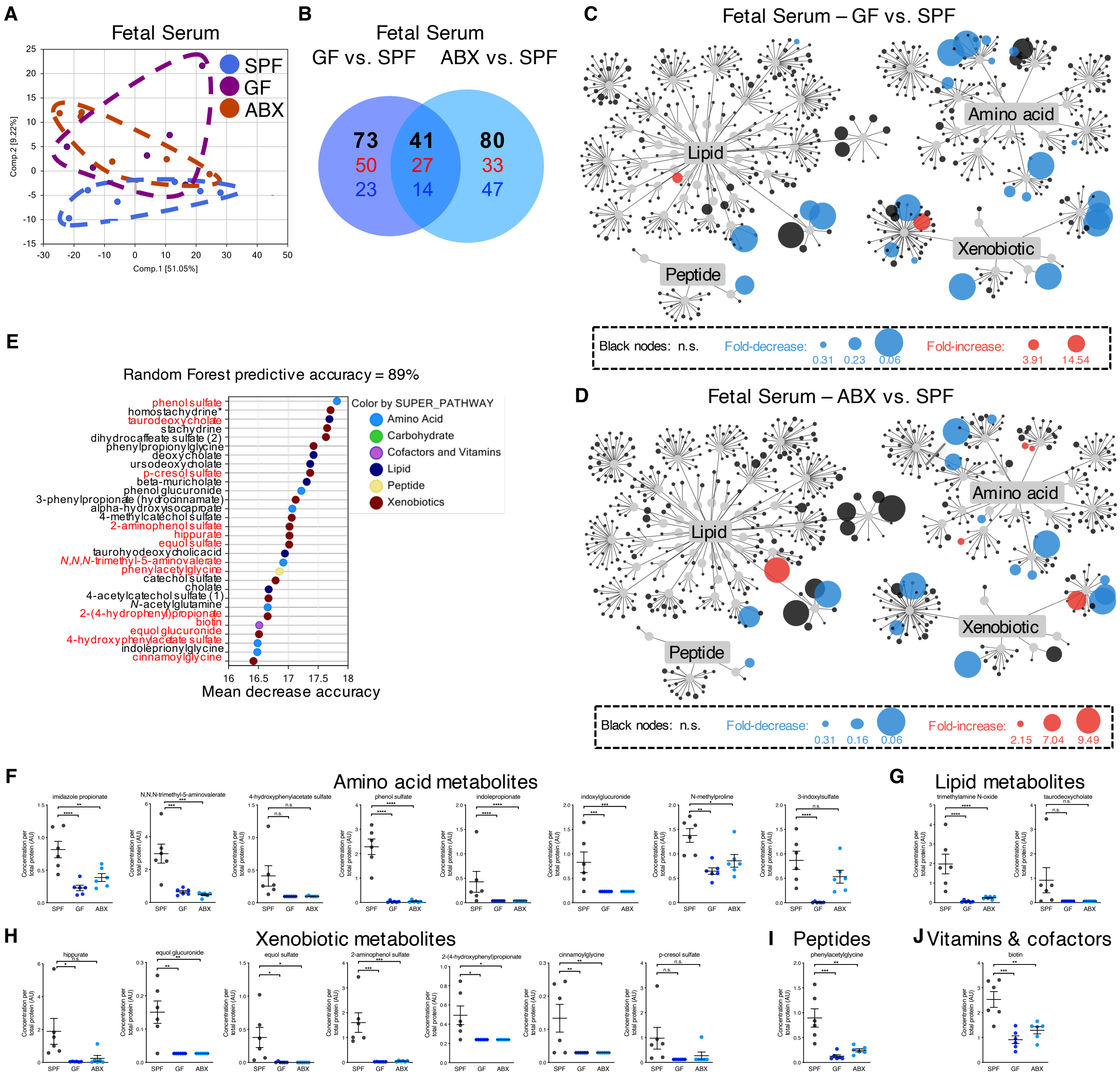
A
Fetal Serum
SPF
GF
ABX
Comp.2 [9.22%]
Comp.1 [51.05%]
B
Fetal Serum
GF vs. SPF
ABX vs. SPF
73
50
23
41
27
14
80
33
47
C
Fetal Serum – GF vs. SPF
Lipid
Amino acid
Xenobiotic
Peptide
Black nodes: n.s.
Fold-decrease: 0.31 0.23 0.06
Fold-increase: 3.91 14.54
D
Fetal Serum – ABX vs. SPF
Lipid
Amino acid
Xenobiotic
Peptide
Black nodes: n.s.
Fold-decrease: 0.31 0.16 0.06
Fold-increase: 2.15 7.04 9.49
E
Random Forest predictive accuracy = 89%
phenol sulfate
homostachydrine*
taurodeoxycholate
stachydrine
dihydrocaffeate sulfate (2)
phenylpropionylglycine
deoxycholate
ursodeoxycholate
p-cresol sulfate
beta-muricholate
phenol glucuronide
3-phenylpropionate (hydrocinnamate)
alpha-hydroxyisocaproate
4-methylcatechol sulfate
2-aminophenol sulfate
hippurate
equol sulfate
taurohyodeoxycholic acid
N,N,N-trimethyl-5-aminovalerate
phenylacetylglycine
catechol sulfate
cholate
4-acetylcatechol sulfate (1)
N-acetylglutamine
2-(4-hydrophenyl)propionate
biotin
equol glucuronide
4-hydroxyphenylacetate sulfate
indolepropionylglycine
cinnamoylglycine
Mean decrease accuracy
Color by SUPER_PATHWAY
Amino Acid
Carbohydrate
Cofactors and Vitamins
Lipid
Peptide
Xenobiotics
F
Amino acid metabolites
imidazole propionate
N,N,N-trimethyl-5-aminovalerate
4-hydroxyphenylacetate sulfate
phenol sulfate
indolepropionate
indoxylglucuronide
N-methylproline
3-indoxylsulfate
G
Lipid metabolites
trimethylamine N-oxide
taurodeoxycholate
H
Xenobiotic metabolites
hippurate
equol glucuronide
equol sulfate
2-aminophenol sulfate
2-(4-hydroxyphenyl)propionate
cinnamoylglycine
p-cresol sulfate
I
Peptides
phenylacetylglycine
J
Vitamins & cofactors
biotin
Concentration per total protein (AU)
SPF GF ABX

Fig. S6: The maternal microbiome modulates the fetal serum metabolome. (A) Principal component analyses of E14.5 fetal serum (SPF (n = 6), GF (n = 6) and ABX (n = 6)). (**B)** Significantly altered metabolites (black text), both downregulated (red text) and upregulated (blue text) in litters represented in Fig. S3B. (**C)** Global metabolite profile changes in GF fetal serum relative to SPF fetal serum. (**D)** Global metabolite profile changes in ABX fetal serum relative to SPF fetal serum. **(E)** Random Forest analysis (predictive accuracy of 89%) identifying the top 30 fetal serum metabolites that distinguish samples as GF or ABX, relative to SPF (metabolites used for *in vivo* supplementation labeled with red text). (**F)** Relative concentrations of amino acid metabolites used for *in vivo* metabolite supplementation from litters represented in Fig. S3A-B. (**G)** Relative concentrations of lipid metabolites used for *in vivo* metabolite supplementation from litters represented in Fig. S3A-B. (**H)** Relative concentrations of xenobiotic metabolites used for *in vivo* metabolite supplementation from litters represented in Fig. S3A-B. (**I)** Relative concentration of peptide metabolites used for *in vivo* metabolite supplementation from litters represented in Fig. S3A-B. (**J)** Relative concentration of vitamin & cofactor metabolites used for *in vivo* metabolite supplementation from litters represented in Fig. S3A-B. Statistical comparisons for all but Fig. S7A and S7E are based on unadjusted $P < 0.05$. Data represent mean ± SEM; statistics were performed with one-way ANOVA with Tukey *post hoc* test. *$P < 0.05$; **$P < 0.01$; ***$P < 0.001$; ****$P < 0.0001$.

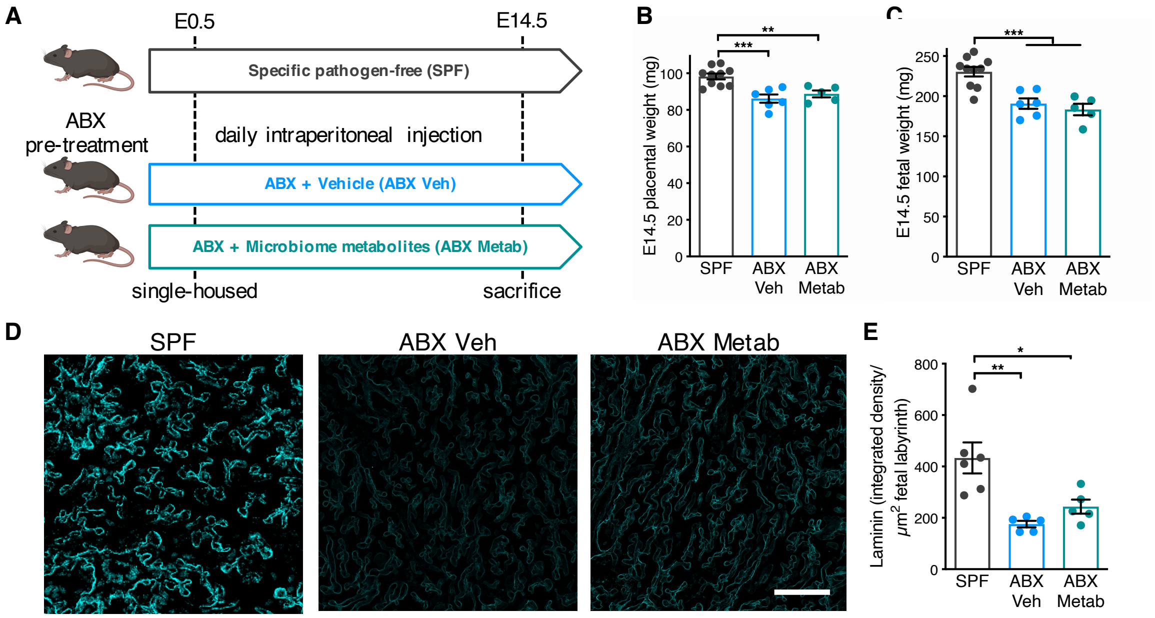

Fig. S7: Select microbiome-dependent metabolites do not rescue placental growth and vascular impairments induced by maternal microbiome depletion. (A) Schematic for microbiome-dependent metabolite supplementation in ABX mice. (**B)** E14.5 placental weights by litter average (SPF (*n* = 10; same as shown in Fig. 1A), ABX Veh (*n* = 6) and ABX Metab (*n* = 5)). **(C)** E14.5 fetal weights for litter averages shown in Fig. S4B. (**D)** Representative images of laminin-stained E14.5 placental labyrinth from SPF (same as in Fig. 2A), ABX Veh and ABX Metab litters. (**E)** Quantification of raw integrated density of laminin intensity, normalized to fetal labyrinth total area (SPF (*n* = 6; same as shown in Fig. 2C), ABX Veh (*n* = 5) and ABX Metab (*n* = 5)). Data represent mean ± SEM; statistics were performed with one-way ANOVA with Tukey *post hoc* test. *$P < 0.05$; **$P < 0.01$; ***$P < 0.001$.

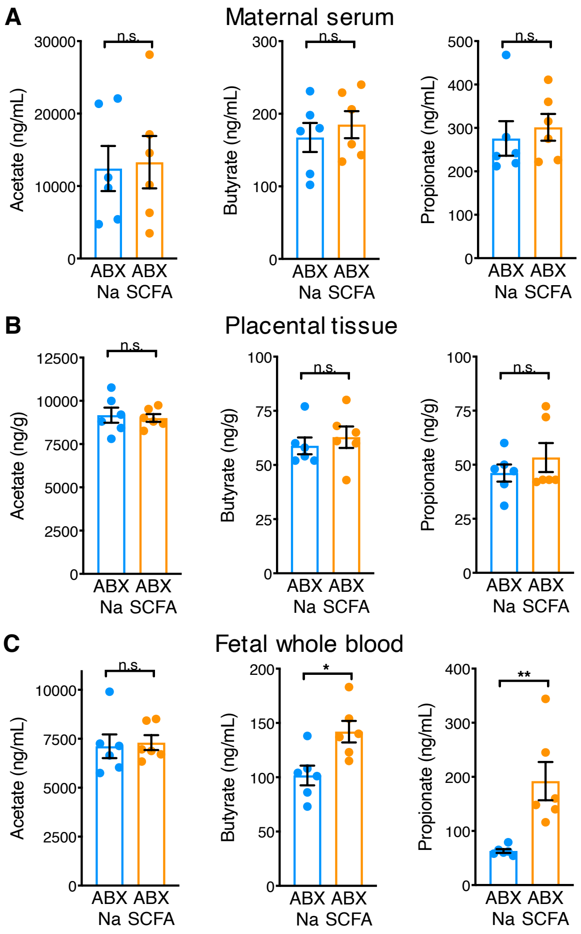

Fig. S8: Intragastric SCFA administration increases fetal blood butyrate and propionate in ABX-treated dams. (A) Quantification of E14.5 maternal serum acetate, butyrate and propionate concentrations, respectively (ABX Na (n = 6) and ABX SCFA (n = 6). (B) Quantification of E14.5 whole placental tissue acetate, butyrate and propionate concentrations, respectively (ABX Na (n = 6) and ABX SCFA (n = 6). (C) Quantification of E14.5 fetal whole blood acetate, butyrate and propionate concentrations, respectively (ABX Na (n = 6) and ABX SCFA (n = 6). Data represent mean ± SEM; statistics were performed with student's t test with Tukey *post hoc* test. *$P < 0.05$; **$P < 0.01$.

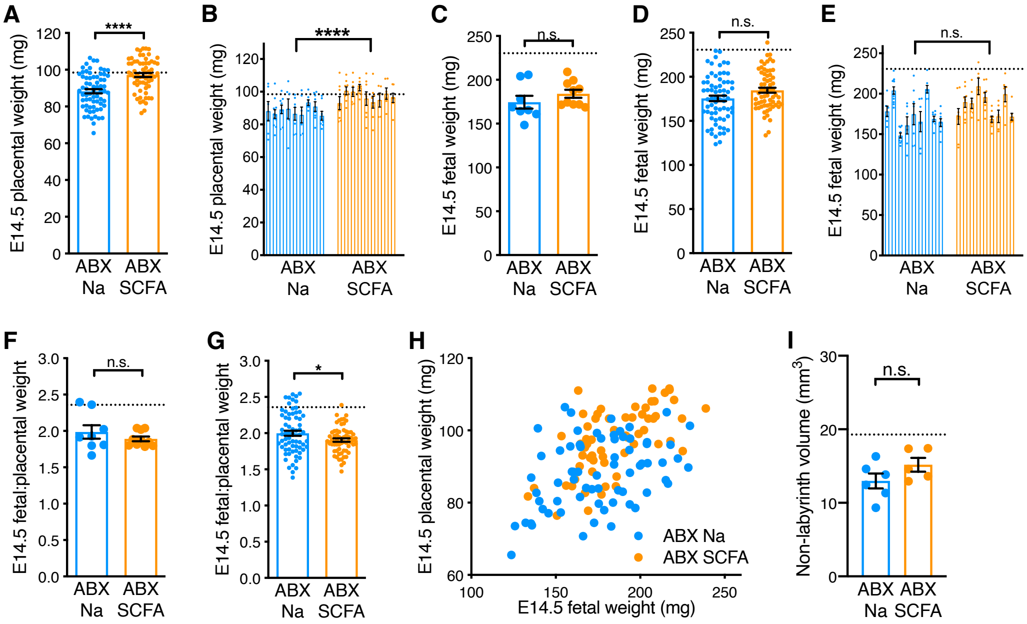

Fig. S9: SCFA supplementation does not alter E14.5 fetal growth in microbiota-depleted dams. (A) E14.5 placental weights for each individual from litters shown in Fig. 3A (ABX Na (n = 68) and ABX SCFA (n = 63); hashed line represents SPF litter average value shown in Fig. 1B). **(B)** Individual E14.5 placental weights used for 1-way nested statistical analysis from litters shown in Fig. S5A (ABX Na (n = 68) and ABX SCFA (n = 63). **(C)** E14.5 fetal weights by litter average for litters shown in Fig. S5A (hashed line represents SPF litter average value shown in Fig. S2B). **(D)** E14.5 fetal weights for each individual from litters shown in Fig. S5A (hashed line represents SPF litter average value shown in Fig. S2C). **(E)** Individual E14.5 fetal weights used for 1-way nested statistical analysis from litters shown in Fig. S5A (ABX Na (n = 68) and ABX SCFA (n = 63). **(F)** E14.5 fetal to placental weight ratios by litter average for litters shown in Fig. S5A-C (hashed line represents SPF litter average value shown in Fig. S2F). **(G)** E14.5 individual fetal to placental weight ratios from litters shown in Fig. S5D (hashed line represents SPF litter average value shown in Fig. S2G). **(H)** Linear correlation of E14.5 placental to fetal weight ratios for individual conceptuses shown in Fig. S5A-C. **(I)** Quantification of E14.5 non-labyrinth placental volumes from µCT reconstructions by litter average (ABX Na (n = 6) and ABX SCFA (n = 5); hashed line represents SPF litter average value shown in Fig. S3L). Means ± SEM (error bars) plotted; statistics were performed with student's t test (litter averages) or with 1-way nested with Sidak multiple comparisons correction (individual conceptuses). . *P < 0.05; ****P < 0.0001.

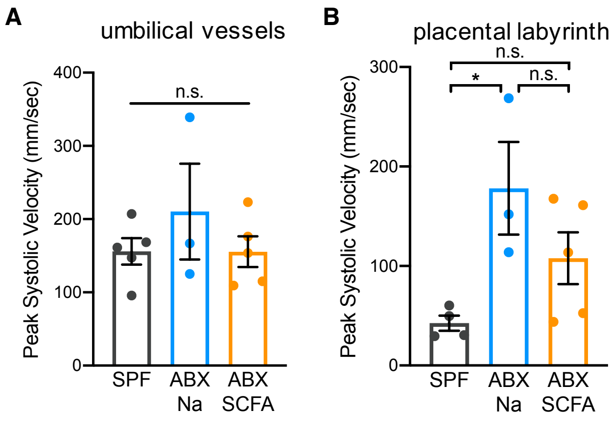

Fig. S10: Maternal SCFA administration partially alleviates impairments in placental labyrinth vascular flow induced by maternal microbiome depletion. (A) Quantification of E14.5 peak systolic velocity in umbilical vessels (SPF (n = 5), ABX Na (*n* = 3) and ABX SCFA (*n* = 5). (**B)** Quantification of E14.5 peak systolic velocity in placental labyrinth from litters shown in SXA (SPF (n = 5), ABX Na (*n* = 3) and ABX SCFA (*n* = 5). Data represent mean ± SEM; statistics were performed with one-way ANOVA with Tukey *post hoc* test. $^*P < 0.05$.

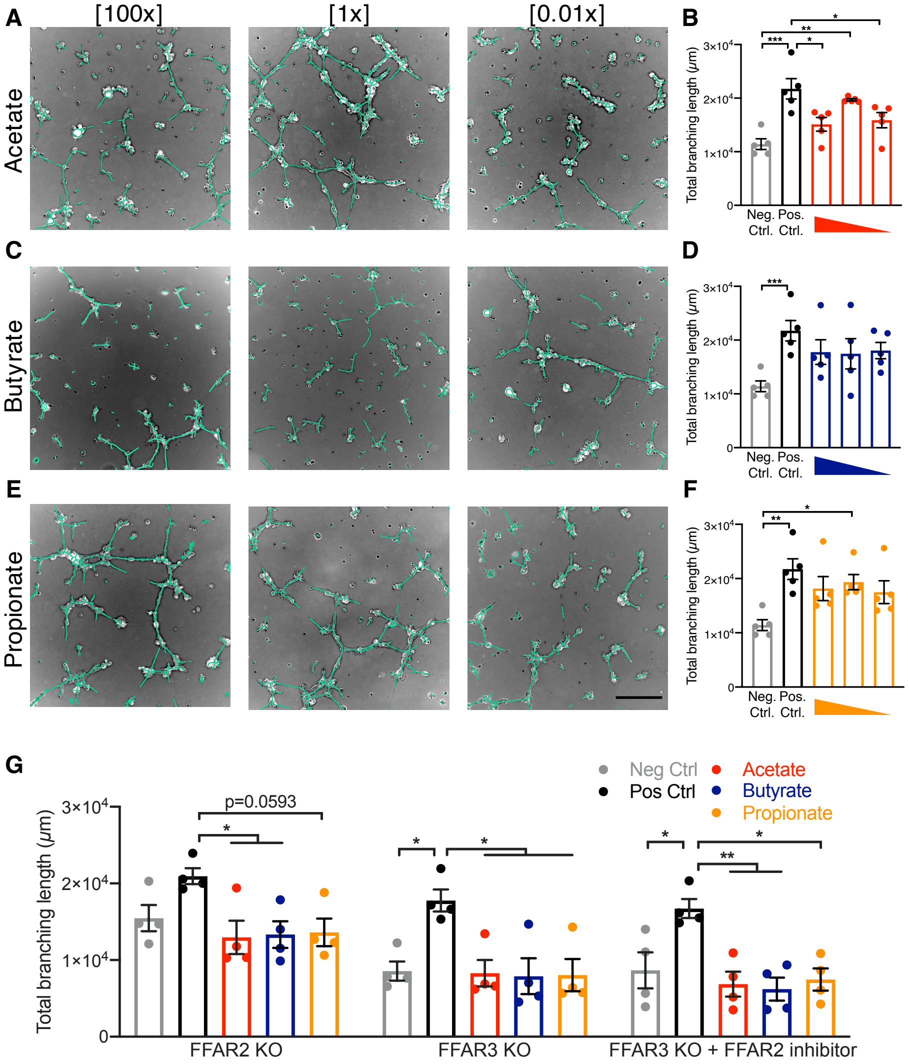

Fig. S11: SCFAs promote HUVEC tube formation *in vitro.* **(A)** Representative images for HUVECs treated with 4 mM, 40 μM and 0.4 μM acetate, respectively. **(B)** Quantification of acetate-supplemented HUVEC tube formation assays. **(C)** Representative images for HUVECs treated with 500 μM, 5 μM and 0.05 μM butyrate, respectively. **(D)** Quantification of propionate-supplemented HUVEC tube formation assays. **(E)** Representative images for HUVECs treated with 500 μM, 5 μM and 0.05 μM propionate, respectively. **(F)** Quantification of butyrate-supplemented HUVEC tube formation assays. **(G)** Quantification of tube formation assays from FFAR2KO HUVECs, FFAR3KO HUVECs and FFAR3KO + FFAR2 inhibitor-treated HUVECs, respectively, treated with 40 μM acetate, 5 μM butyrate or 5 μM propionate. Scale bar = 250 μm. Data represent mean ± SEM; statistics were performed with one-way ANOVA with Tukey *post hoc* test. *$P < 0.05$; **$P < 0.01$; ***$P < 0.001$.

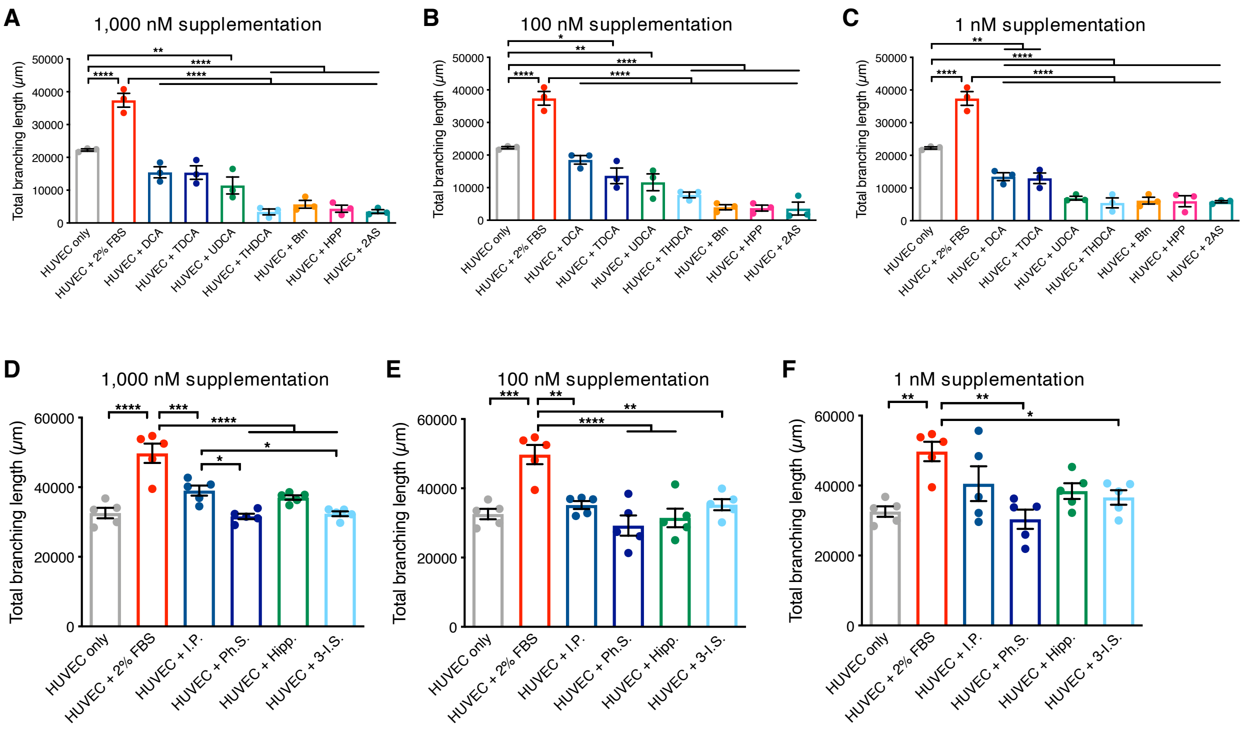

Fig. S12: Select microbiome-dependent metabolites do not promote HUVEC tube formation *in vitro*. (A) Quantification of HUVEC branching length in non-supplemented media (HUVEC only, negative control), 2% FBS-supplemented media (positive control), and supplementation with 1,000 nM deoxycholate (DCA), taurodeoxycholate (TDCA), ursodeoxycholate (UDCA), taurohyodeoxycholate (THDCA), biotin (Btn), 2-(4-hydrophenyl)propionate (HPP) and 2-aminophenol sulfate (2AS), respectively. **(B)** Quantification of HUVEC branching length in non-supplemented media, 2% FBS-supplemented media, and supplementation with 100 nM DCA, TDCA, UDCA, THDCA, Btn, HPP and 2AS, respectively. **(C)** Quantification of HUVEC branching length in non-supplemented media, 2% FBS-supplemented media, and supplementation with 1 nM DCA, TDCA, UDCA, THDCA, Btn, HPP and 2AS, respectively. **(D)** Quantification of HUVEC branching length in non-supplemented media, 2% FBS-supplemented media, and supplementation with 1,000 nM imidazole propionate (I.P.), phenol sulfate (Ph.S.), hippurate (Hipp.) and 3-indoxyl sulfate (3-I.S.), respectively. **(E)** Quantification of HUVEC branching length in non-supplemented media, 2% FBS-supplemented media, and supplementation with 100 nM imidazole propionate (I.P.), phenol sulfate (Ph.S.), hippurate (Hipp.) and 3-indoxyl sulfate (3-I.S.), respectively. **(F)** Quantification of HUVEC branching length in non-supplemented media, 2% FBS-supplemented media, and supplementation with 1 nM imidazole propionate (I.P.), phenol sulfate (Ph.S.), hippurate (Hipp.) and 3-indoxyl sulfate (3-I.S.), respectively. Data represent mean ± SEM; statistics were performed with one-way ANOVA with Tukey *post hoc* test. $*P < 0.05$; $**P < 0.01$; $***P < 0.001$; $****P < 0.0001$.

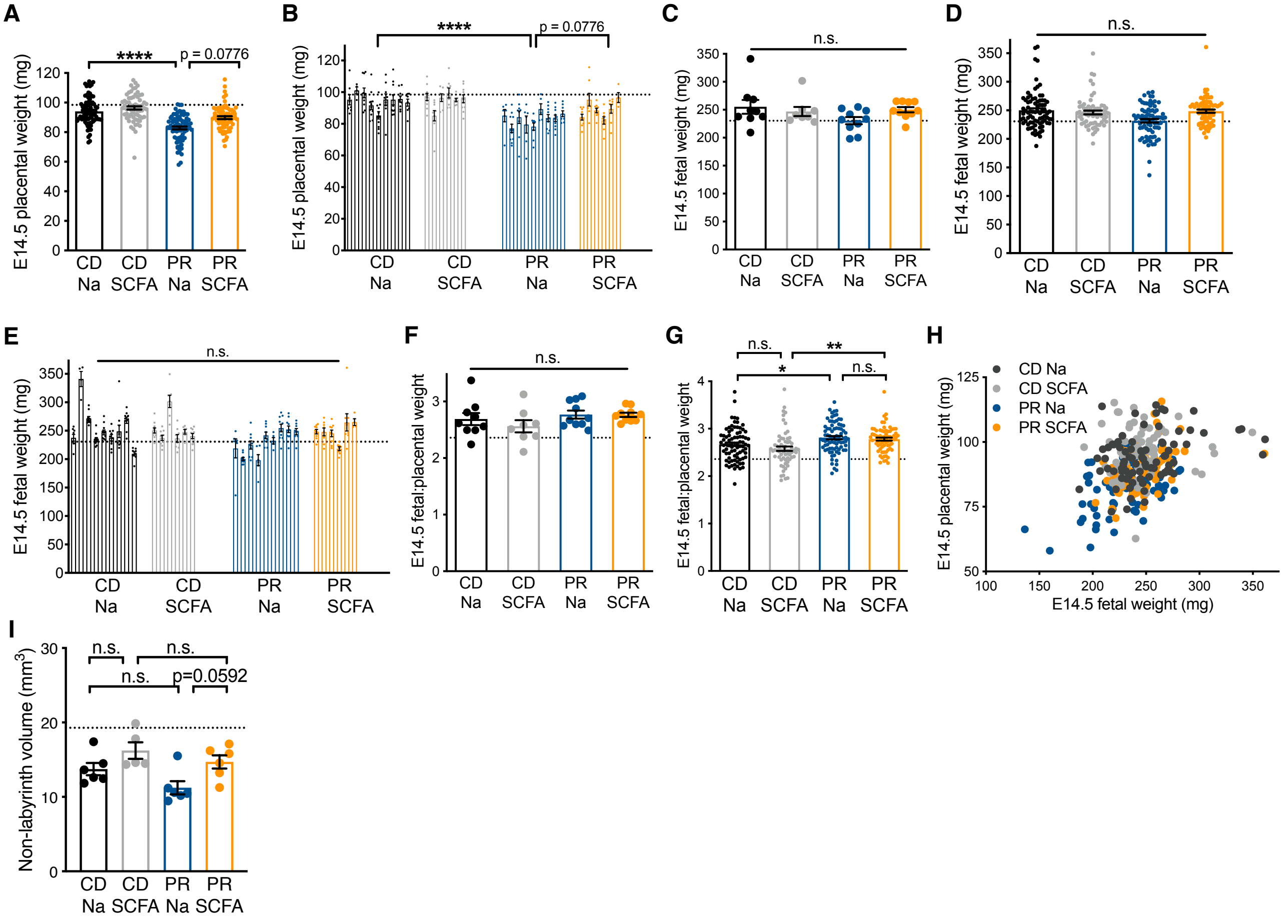

Fig. S13: Maternal SCFA supplementation modestly increases E14.5 fetal growth in protein-restricted dams. (A) E14.5 placental weights for each individual from litters shown in Fig. 4A (CD Na (n = 80), CD SCFA (n = 65), PR Na (n = 81) and PR SCFA (n = 72); hashed line represents SPF litter average value shown in Fig. 1B). **(B)** Individual E14.5 placental weights used for 1-way nested statistical analysis from litters shown in Fig. S8A (CD Na (n = 80), CD SCFA (n = 65), PR Na (n = 81) and PR SCFA (n = 72)). **(C)** E14.5 fetal weights by litter average for litters shown in Fig. S8A (hashed line represents SPF litter average value shown in Fig. S2B). **(D)** E14.5 fetal weights for each individual from litters shown in Fig. S8A (hashed line represents SPF litter average value shown in Fig. S2C). **(E)** Individual E14.5 fetal weights used for 1-way nested statistical analysis from litters shown in Fig. S8D (CD Na (n = 80), CD SCFA (n = 65), PR Na (n = 81) and PR SCFA (n = 72)). **(F)** E14.5 fetal to placental weight ratios by litter average for litters shown in Fig. S5A-C (hashed line represents SPF litter average value shown in Fig. S2F). **(G)** E14.5 individual fetal to placental weight ratios from litters shown in Fig. S5D (hashed line represents SPF litter average value shown in Fig. S2G). **(H)** Linear correlation of E14.5 placental to fetal weight ratios for individual conceptuses shown in Fig. S5A-C. **(I)** Quantification of E14.5 non-labyrinth placental volumes from µCT reconstructions by litter average (CD Na (n = 6), CD SCFA (n = 5), PR Na (n = 6) and PR SCFA (n = 5); hashed line represents SPF litter average value shown in Fig. S3L). Data represent mean ± SEM; statistics were performed with two-way ANOVA with Tukey *post hoc* test (litter averages) or with 1-way nested with Sidak multiple comparisons correction (individual conceptuses). $^{**}P < 0.01$; $^{****}P < 0.0001$.

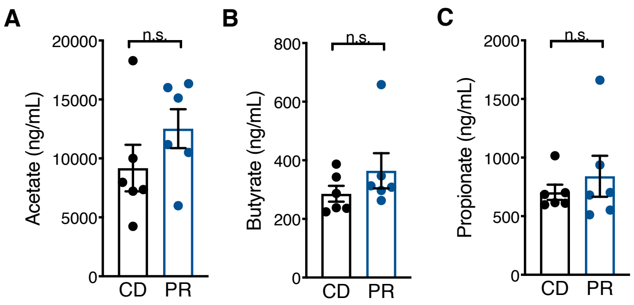

Fig. S14: Maternal serum SCFA concentrations are not affected in dietary maternal protein restriction model. (A) Quantification of E18.5 maternal serum acetate concentrations (CD (n = 6) and PR (n = 6)). (**B)** Quantification of E18.5 maternal serum butyrate concentrations (CD (n = 6) and PR (n = 6)). (**C)** Quantification of E18.5 maternal serum propionate concentrations (CD (n = 6) and PR (n = 6)). Data represent mean ± SEM; statistics were performed with student's t test with Tukey *post hoc* test.

Table S1 (separate file): Metabolites in E14.5 SPF, GF and ABX fetal blood

Table S2 (separate file): Metabolites used for in vivo supplementation in ABX dams

Table S3 (separate file): Metabolite concentrations used in HUVEC branching tube formation assays

Table S4 (separate file): 16S rRNA gene sequencing of fecal microbiota from ABX, Non Abs and Abs dams on E14.5

Table S5 (separate file): Compositions of mouse diets

Data S1 to S18 (separate file): Raw input data for each graph of all figures.
